# May phytophenolics alleviate aflatoxins-induced health challenges? A holistic insight on current landscape and future prospects

**DOI:** 10.3389/fnut.2022.981984

**Published:** 2022-10-28

**Authors:** Hassan Rasouli, Fatemeh Dehghan Nayeri, Reza Khodarahmi

**Affiliations:** ^1^Medical Biology Research Center (MBRC), Kermanshah University of Medical Sciences, Kermanshah, Iran; ^2^Department of Biotechnology, Faculty of Agricultural and Natural Sciences, Imam Khomeini International University (IKIU), Qazvin, Iran

**Keywords:** Climate change, Aflatoxin, Phytophenolics, Cancer, Diabetes, Alzheimer's disease, Oxidative stress, Inflammation

## Abstract

The future GCC-connected environmental risk factors expedited the progression of nCDs. Indeed, the emergence of AFs is becoming a global food security concern. AFs are lethal carcinogenic mycotoxins, causing damage to the liver, kidney, and gastrointestinal organs. Long-term exposure to AFs leads to liver cancer. Almost a variety of food commodities, crops, spices, herbaceous materials, nuts, and processed foods can be contaminated with AFs. In this regard, the primary sections of this review aim to cover influencing factors in the occurrence of AFs, the role of AFs in progression of nCDs, links between GCC/nCDs and exposure to AFs, frequency of AFs-based academic investigations, and world distribution of AFs. Next, the current trends in the application of PPs to alleviate AFs toxicity are discussed. Nearly, more than 20,000 published records indexed in scientific databases have been screened to find recent trends on AFs and application of PPs in AFs therapy. Accordingly, shifts in world climate, improper infrastructures for production/storage of food commodities, inconsistency of global polices on AFs permissible concentration in food/feed, and lack of the public awareness are accounting for a considerable proportion of AFs damages. AFs exhibited their toxic effects by triggering the progression of inflammation and oxidative/nitrosative stress, in turn, leading to the onset of nCDs. PPs could decrease AFs-associated oxidative stress, genotoxic, mutagenic, and carcinogenic effects by improving cellular antioxidant balance, regulation of signaling pathways, alleviating inflammatory responses, and modification of gene expression profile in a dose/time-reliant fashion. The administration of PPs alone displayed lower biological properties compared to co-treatment of these metabolites with AFs. This issue might highlight the therapeutic application of PPs than their preventative content. Flavonoids such as quercetin and oxidized tea phenolics, curcumin and resveratrol were the most studied anti-AFs PPs. Our literature review clearly disclosed that considering PPs in antioxidant therapies to alleviate complications of AFs requires improvement in their bioavailability, pharmacokinetics, tissue clearance, and off-target mode of action. Due to the emergencies in the elimination of AFs in food/feedstuffs, further large-scale clinical assessment of PPs to decrease the consequences of AFs is highly required.

## Prologue

Nowadays, the estimations predict that the global demand for intensified food production has been increased, and the statistics are expected to be doubled by 2050 (1, 2). Providing safe foods to nurture the world population requires a significant improvement in crop cultivation systems, plant breeding techniques, and the development of climate-smart crops (3). GCC and environmental forces are two determinant factors to influence crops' sustainable growth. GCC influences crop production practices in sophisticated modes (3, 4). Numerous studies have been addressed the direct and indirect effects of GCC on world food demand and agricultural systems (5, 6). Accordingly, modification of cultivation systems and increasing the susceptibility of crops to future climate are typical direct effects of climate change. From a large-scale perspective, affecting the world economy, food demand, and distributions of incomes are indirect effects of climate change on world societies (4).

Studies have shown that changes in climate humidity, temperature, and precipitation patterns are associated with the outbreak of some invasive fungal pathogens (7). The uncontrolled growth of these fungi affects the quality and quantity of crop yield, stored foodstuffs, grains, processed foods, and herbaceous products (8). Studies highlighted the possible health risks linked to fungal toxins to the human body (8). The APF including *Aspergillus, Fusarium*, and *Penicillium*, are well-known mycotoxigenic fungal species with potentially fatal effects on human and animal health (9). The occurrence of AFs in food commodities depends on environmental factors such as air temperature, humidity, CO_2_ levels, pH, susceptibility of foods to contaminations, and improper harvest and storage of food products (10, 11). According to scientific reports, nearly 25% of global food supplies are contaminated with AFs, making them a serious issue of concern for world nations' health (12). AFs are health hazardous environmental risk factors in the onset of liver cancer, kidney failure, and gastrointestinal problems (13), in turn, their health consequences depend on duration of exposure and enzymatic/genetic alterations in target organs (14).

Poverty, hunger and contaminated foods are the foremost health risk factors to threaten human life. Numerous studies suggested that mycotoxins, including aflatoxins, ergot alkaloids, ochratoxins, trichothecenes, zearalenone, and fumonisins, are among the most lethal naturally occurring toxins (15, 16). Characterization and elimination of mycotoxins in food/feed items require specific technical and analytical methods (17). Developed countries implemented strict regulatory gates to control and monitor the occurrence of AFs in import/export sites to reduce the health complications of these toxins (18). In contrast, many people in low-income countries are at risk of long-term exposure to AFs, resultantly this might increase the progression of different cancers in these locations (19).

Studies have shown that natural products (e.g., PPs, berberine, plant extracts, polysaccharides, etc.) can reduce the toxicity and production of AFs (20, 21). PPs are a large heterogeneous group of secondary plant metabolites, display a wide range of biological activities, and are substantially studied for their anticancer activities (22–24). The regular consumption of PPs is associated with lower risks for developing cardiovascular diseases, obesity, DM, cancer, stroke, and AD (23–26), though this finding requires further well-designed clinical validations. Global interest in PPs studies to alleviate AFs health consequences has been increased during the past decades. Preliminary studies showed that PPs can directly and/or indirectly alter the possible toxicity effects of AFs in complicated ways (27, 28). However, the molecular mechanisms underlying PPs effects on AFs toxicity in the human body are still not understood comprehensively, and scientific efforts are ongoing to find out the health-promoting content of PPs against AFs.

In this regard, this review aims to summarize recent findings on AFs and the application of PPs in alleviating AFs end effects. Due to the strategic roles of antioxidant phytochemicals in preventing human chronic diseases (29), and beneficial effects of PPs in the cornerstone of therapeutic programs (30, 31), we have followed five goals herein: (1) providing a comprehensive insight on the association of GCC and the occurrence of AFs; (2) characterizing the most potent anti-AFs phenolics; (3) role of AFs in the onset of nCDs; (4) understanding anti-AFs mechanism of actions of PPs; and (5) addressing the current gaps regarding the large-scale application of PPs for clinical applications.

## Literature search strategy

Scientific databases including Scopus, PubMed, Google Patents, and Scholar were separately searched to find relevant papers using keywords such as “aflatoxin or human diseases,” “aflatoxin B1/B2/G1/G2/M1,” “aflatoxin and GMO,” “aflatoxin and diabetes,” “aflatoxin and cancer,” “aflatoxin B1 or chronic diseases,” “aflatoxin or transcriptome,” “aflatoxin and/or Alzheimer's,” “aflatoxin and flavonoids,” “aflatoxin or polyphenols,” “aflatoxin and/or stilbenes,” “aflatoxin and curcumin,” “aflatoxin and/or epigenetic,” “aflatoxin and/or plant extract,” “aflatoxin and/or crops,” “aflatoxin M1 and/or foodstuff,” aflatoxin and/or spices,” “aflatoxin and climate change,” “aflatoxin and/or temperature,” “aflatoxin and/or CO_2_ levels.”

The outputs of searches were used to generate bibliometric network using VOSviewer software (32). More than 20,000 papers (published from 1990 to 2021) were appeared in search outputs. To unify the results, we compared the outcomes together, thus the redundant papers with similar title or content were deleted from search outputs. To interpret the statistical output of literature searches, we used Scopus data to illustrate the relevant graphs. We also used Cytoscape (ClueGo module) software (33) to construct simplified protein-protein interaction networks where it was needed. Because the exact binding modes of all AFB1 metabolites into human serum albumin (HSA) were not available in the literature, we used AutoDock Vina tools (34) to generate the expected interactions. The Protein-Imager software (35) was recruited to inspire some of graphical illustrations.

## General overview of AFs chemistry

Fungal AFs including B1(AFB1), B2(AFB2), M1(AFM1), G1(AFG1), and G2(AFG2) are well-known AFs in contaminated crops, foods, dairy products, herbal materials, spices, and processed foods (19, 36). AFs showed mutagenic, carcinogenic, hepatogenic, teratogenic, and immunosuppressive toxicological properties. The toxic properties of AFs depend on alteration of enzymatic activity, modification of gene expression patterns, epigenetics changes, and dysregulation of signaling pathways (11–13, 37, 38).

**Figure 1:**
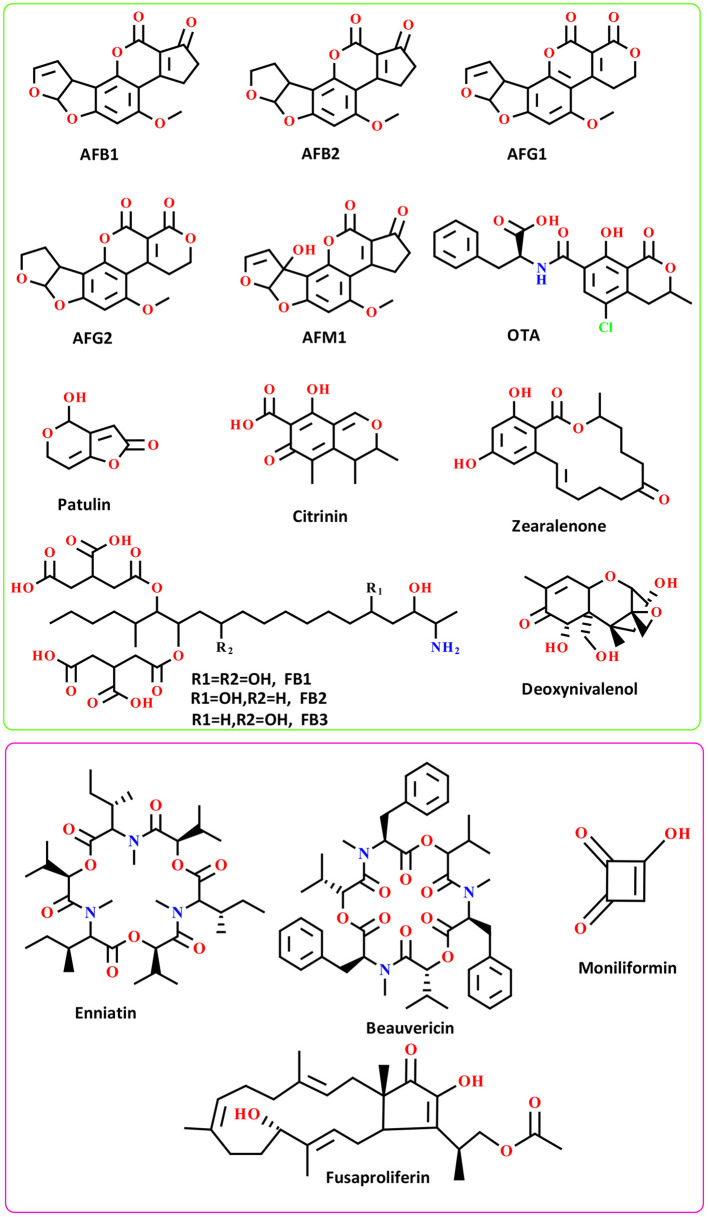
The chemical structure of well-known AFs (green box) and emerging mycotoxins (pink box); FB: Fumonisins B.

Chemically, AFs can be classified into two main groups including difuran-coumarin lactones (AFG1/2) and difuran-cyclo-pentanones (AFB1/2 and AFM1/2) (39, 40). These fungal toxins displayed a “CHO” molecular formula with a different number of H/O atoms. The molecular weight of AFs ranges from 312 to 330 g·mol^−1^. AFs displayed a colorful fluorescent pattern under the UV light. In this respect, AFB1/2 showed a blue color while AFGs displayed green color (39, 40) (Figure 1). To date, more than 20 different AFs have been identified with moderate to high toxicity effects on the human body (40). The evidence suggests that AFs are potent carcinogenic toxins with potential side effects on human liver organs and tissues (37).

The laboratory analysis of AFs identified several metabolized AFs derivatives that rarely found in the human body (40). The most abundant AFs derivatives found in human and animal bodies are AFM1/2 metabolites-the derivatives of AFB1 metabolism in the liver (41). These AFs are widely found in milk and milk-based products, and recently received much attention from the literature due to their potential health problems for consumers of dairy products (41). Studies have shown that AFs are thermostable substances, therefore elevated temperature might not destroy these mycotoxins (40). The toxicity content of AFs is in the order of AFB1 > AFG1 > AFB2 > AFG2 (42). These mycotoxins have been considered as group I highly carcinogenic substances by IARC (40, 43). No only structural features of well-known AFs, but also the chemistry of emerging toxins mainly produced by *Fusarium* species should be taken into account because of their potential to outbreak and influence the future food safety and security (44). For more details, the chemistry and distribution of emerging mycotoxins have been reviewed by Gruber-Dorninger and colleagues (45).

## The upcoming GCC and future AFs contamination risks

The GCC is becoming a serious concern, and being the future driver of food safety and security (46). The expanded worldwide industrial activities and the increasing rate of world population are the two of the most crucial components of countries' climate modification (47–49). The outbreak of invasive plant pests/pathogens, soil erosion, drought, erratic rainfall patterns, GW, salinity, crop cultivation failure, shortage of irrigation water, and reduction in the fertility of arable soils are the foremost consequences of the GCC (47, 48, 50, 51).

Studies disclosed that GCC induces the occurrence of plant fungal pathogens by providing appropriate environmental requirements for their growth and development (52, 53). While elevated temperatures and irregular precipitation patterns can significantly reduce crop yield, the growing evidence suggests that these climatic consequences also triggered the production of AFs at the beginning of crops growing seasons (53). The emerging evidence postulates that under at least +2 to +5°C climate temperature increase, maize and wheat crops might prone to higher levels of AFs contamination (46). Studies also demonstrated that APF tolerated a wide range of temperatures, resultantly enabled these fungi to grow easily in the production/storage sites of crops (54).

The GCC might support the growth and development of APF in crop production sites (7, 55). The overgrowth of these fungi leads to a rapid expansion of spores in the environment, increases AFs contaminants level, ultimately leading to health impacts on consumers (7, 54). Due to the increased universal demand for food, the GCC intensifies crop cultivation and production, in turn, might lead to the establishment of single-crop cultivation systems (56). Additionally, underestimating of crop rotation and plantation of susceptible crops may also increase the risk of future AFs occurrence (57).

Different strategies have been applied to address the GCC in advanced and developing countries (3, 54). These policies mainly focused on improving breeding strategies (3) and recruiting integrated and novel methods to control plant pathogens (58). In contrast, farming systems in undeveloped countries are almost entirely influenced by GCC (1). On such occasions, traditional methods of crop cultivation, harvest, and storage have mainly been followed by local farmers, these may in turn be leading to the exacerbation of APF outbreak (59). Although a variety of sophisticated methods have been employed in developed countries for integrated control of mycotoxin-producing fungi in food and feed (54, 60), the outcomes, however, suggested that these methods were rarely successful to completely reduce the occurrence of AFs (18, 61).

The emerging reports on the GCC demonstrated that GW has begun to occur but the empirical data on how GW might affect crops yield is markedly overlooked (62). Strategically, GW and GCC might alter the distribution of plant pests and diseases, resulted in a significant damage to crop production. The literature suggests that pest-infested crops are prone to AFs contaminants (63). Therefore, predicting the exact roles of GCC on the prevalence of AFs relies the development of accurate models to estimate the future damages of AFs under GCC (64). Presently, mechanistic (65), empirical, and hybrid models in predicting future economic costs of AFs occurrence have been developed in Australia, the USA and some European countries (64) while the lack of comprehensive predictive models in less developed countries (e.g., Africa, Middle-East, Latin America) might decrease the effectiveness of such estimations in preventing future AFs production (64, 66).

Therefore, recruiting predictive models to simulate the occurrence of AFs requires an in-depth knowledge of future AFP-GCC interactions (67) to know where and how these mycotoxins will be emerged in the target production/storage sites (64, 66), which future crops are more susceptible to AFs, and ultimately which future country-specific regulations must be taken into consideration in order to better elimination of AFs production. Indeed, in addressing modeling of future APF-GCC interactions, it is also meritorious to highlight this note that the contemporaneous predictive studies have been validated in limited geographical regions, therefore it is imperative to conduct large-scale multinational investigations for better understanding of GCC impacts on future APF mycoflora and global pattern of AFs distribution (68).

In this regard, Yu et al. reported that global temperature modification has an impact on the prevalence of AFs contaminants (62). According to the given model for simulation of AFs occurrence based on corn phenology, it is believed that some corn grown US states will experience an increased level of AFs occurrence by 2031–2040 (62). On the contrary, this estimation also postulated that under elevated temperatures AFs might be inactivated, therefore, some the US counties might experience lower level of AFs occurrence (62). Correspondingly, other outcomes also suggested that water stress and elevated temperatures are two determinant factors in changing the relative expression pattern of structural genes (*aflD, aflR*) involved in the production of AFB1, leading to higher occurrence of this carcinogenic mycotoxin (67).

Scientists recruited high-throughput multi-omics technologies to assess the impact of GCC on the production of AFB1 (69). The outcomes unraveled that the elevated CO_2_ levels, as a consequence of GW, might alter *aflR* gene expression in AFB1 biosynthetic pathway (69). In another study, researchers simulated climate change condition to investigate how it might influence the growth of *A. carbonarius* and OTA production under elevated temperature/CO_2_ levels (70). The results surprisingly displayed that the interaction between elevated CO_2_ levels and temperature lead to the up-regulation of velvet complex regulatory elements and OTA biosynthetic genes in *A. carbonarius*. This finding suggests that elevated CO_2_/temperature levels are two quintessential factors in increasing the risk of OTA contaminants in grape-based products (70). The outcomes from a similar study also demonstrated that changes in temperature/CO_2_ levels in stored coffee beans and coffee-based media attributed to OTA production in *A. westerdijkiae* compared to *A. carbonarius* (71).

In another study, it was also reported that the GCC-associated factors have differential impacts on AFB1 production in pistachio nuts (7). In a case study conducted on maize grown in Eastern Europe using different climatological models, the estimations predicted that climate change can lead to a probable increase in the occurrence of maize AFB1 and cow's milk AFM1 (72). Other studies on the effects of GW on the occurrence of AFB1 and trichothecenes mycotoxins in wheat and maize crops also suggested that the prevalence of these hazardous mycotoxins is expected to increase as consequences of the future GCC (46, 68). Although little is understood on the effects of future GCC on mycotoxigenic fungi growth and mycotoxins production, developing accurate predictive models to characterize mechanistic interactions of APF with climatological factors will provide a ground for better controlling of these fungi (68).

It is estimated that economic losses due to the occurrence of AFs are between $500 million to $1.6 billion for maize, peanuts and other crops in the USA (73, 74). These obvious economic costs, however, are associated with GCC and its impact on AFs production (73). Another pivotal issue, that is crops grown in low and northern latitudes might negatively or positively deal with future GCC (44). In this regard, the evidence suggests that low latitude regions will be suffered from consistent and negative consequences of GCC compared to the northern regions where its effects may be positive or negative (44).

Considering the relationship between GCC and increased global demand for food, this might threaten the production of certain crops such as maize using the available infrastructures. In this respect, the occurrence of AFs and other mycotoxins is expected to increase on such occasions (75). In spite of the fact that the universal temperature may be rising above the optimum condition for APF, it is pivotal to consider the threat posed by emerging thermotolerant fungi that can produce novel health hazardous toxins (76). Therefore, smart crop breeding for developing resistance against both GCC/GW and APF might be considered as an alternative scenario in managing the reduction of AFs occurrence in food and feed (75).

**Figure 2:**
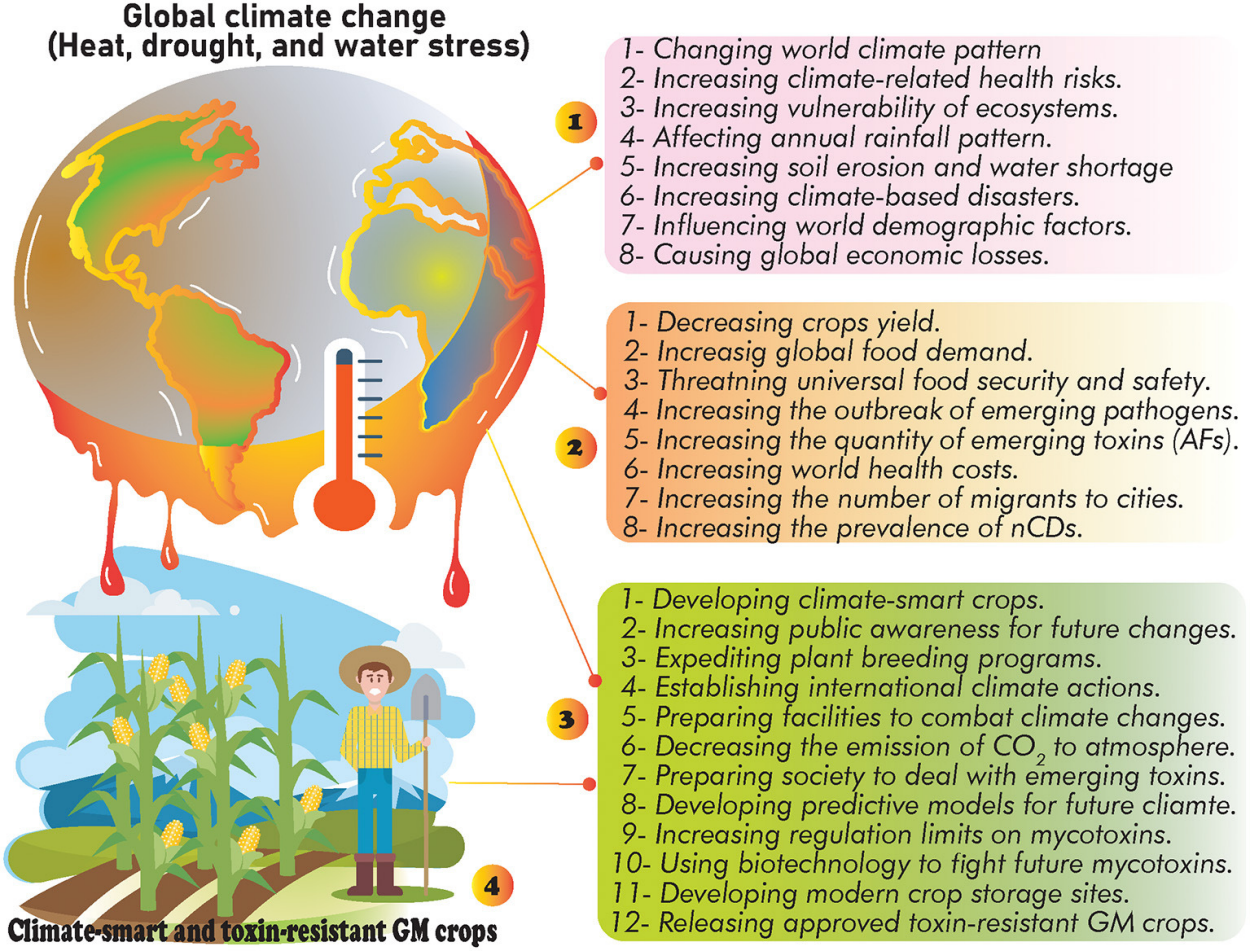
The possible effects of GCC: **(1)** general side effects of GCC, **(2)** consequences of GCC on food security and public health, **(3)** the suggested strategies to deal with future GCC, and **(4)** developing climate-smart crops as alternative way to decrease the occurrence of AFs and neutralize the consequences of future GCC.

In addition to financial losses to animal and agricultural commodities, AFs-contaminated foods are leading to significant clinical costs due to the side effects of long-term exposure to these toxins for the human body (18). However, decreasing milk production, reducing of crops quality, weakening animal immune system, and many other demerits are few examples of AFs financial burden for animal and crop products (18). Various chemical and biological methods have been suggested in controlling AFs-producing molds (60, 77, 78), nevertheless, the evidence suggests that there has been no efficient method to completely eliminate these molds (60, 78, 79). It should be expected that the universal quantity/distribution of AFs will probably be grown under GCC/GW. Therefore, due to the upcoming future GCC, systematic modification of crop cultivation practices, applying crop rotation, developing climate-smart crops, set limits to APF growth in production sites, modernizing crop storage facilities, utilizing modern crop irrigation systems, and increasing public awareness about the association of climate change and AFs risks are influential scenarios to reduce the occurrence of AFs under modified future climate (Figure 2).

Studies have also highlighted the role of GCC in the prevalence of nCDs such as cancer, MetSys, stroke, chronic respiratory disorders, and cardiovascular diseases (80, 81). In this regard, several investigations purported that the future GW/GCC will highly increase exposure levels to GCC-associated health hazardous risk factors (49, 66, 68, 72), consequently leading to higher rates of global deaths (49, 81). As discussed, AFs exposure will probably be growing in the upcoming years owing to a significant modification in countries' climate patterns (82), nevertheless, the current estimations require further validations to address all gaps and challenges in preparing world communities for future changes (68).

The co-occurrence of GCC-associated risk factors offering synergistic effects on human health and the onset of nCDs (83, 84). Therefore, due to the complexity of interactions between GCC/exposure to AFs (and other emerging mycotoxins) (85), and the onset of nCDs (86), it is obligatory to establish country-specific regulations to deal with the upcoming challenges (64) and decrease the global burden of incurable human diseases. While only 2% of global health funds allocated to treat these diseases, the estimations predicted that the number of people affected by nCDs have dramatically been increased over the past decades (81, 87). Therefore, due to the lack of specific international leadership to combat nCDs (81), the increased exposure to AFs and other emerging mycotoxins under countries' climate change will worsen global health status, particularly in low and middle-income countries (88, 89). In this regard, to decrease the economic costs of AFs exposure and the progression of nCDs, the possible threats of future GCC should be taken into account in alleviating the health consequences of AFs risks.

## Increasing world studies on AFs

**Figure 3:**
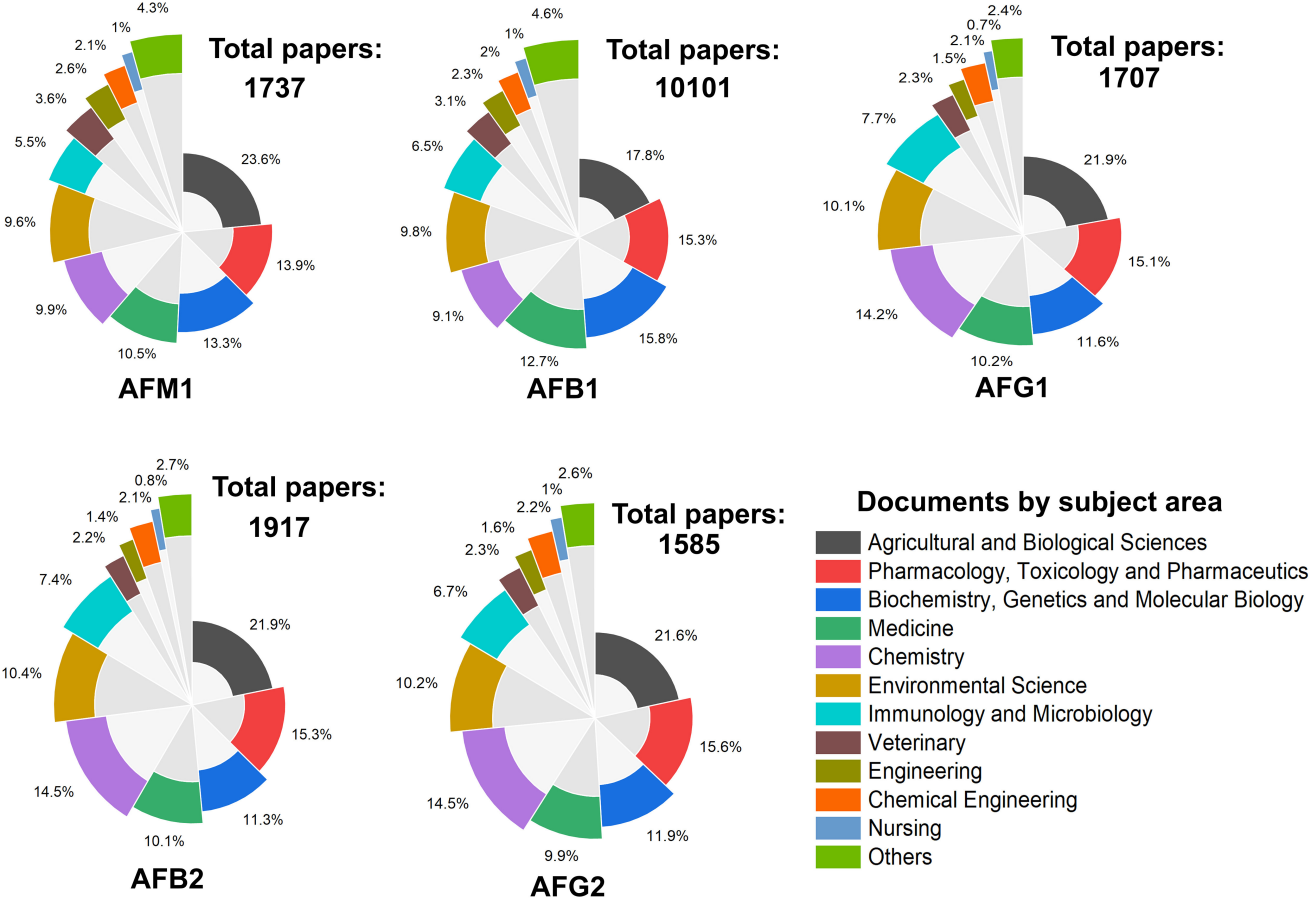
The frequency of studies on AFs metabolites from 1990 to 2021. Each plot represents the number of papers published on searched keywords and the percentage of documents per subject area.

According to the Scopus statistics (https://www.scopus.com/), the global studies on AFs have markedly increased during the past few decades. The literature mining of scientific databases showed that more than 20,000 papers have been published on different classes of AFs from 1990 to 2021. As depicted in Figure 3, the frequencies of studies on different types of AFs metabolites displayed that most of these studies had targeted AFs metabolites such as AFB1/2, AFG1/2, and AFM1. Interestingly, the number of studies on AFM2 was lower than other AFs metabolites during the investigated timeline, and these investigations have increased from 2008 to 2021. Accordingly, a large proportion of scientific studies on AFs were conducted in field of agriculture and biological sciences, followed by biochemistry, medicine, and chemistry, respectively. These frequencies of studies on AFs displayed that these mycotoxins received much attention from academia and clinical sectors due to their health hazardous risks.

**Figure 4:**
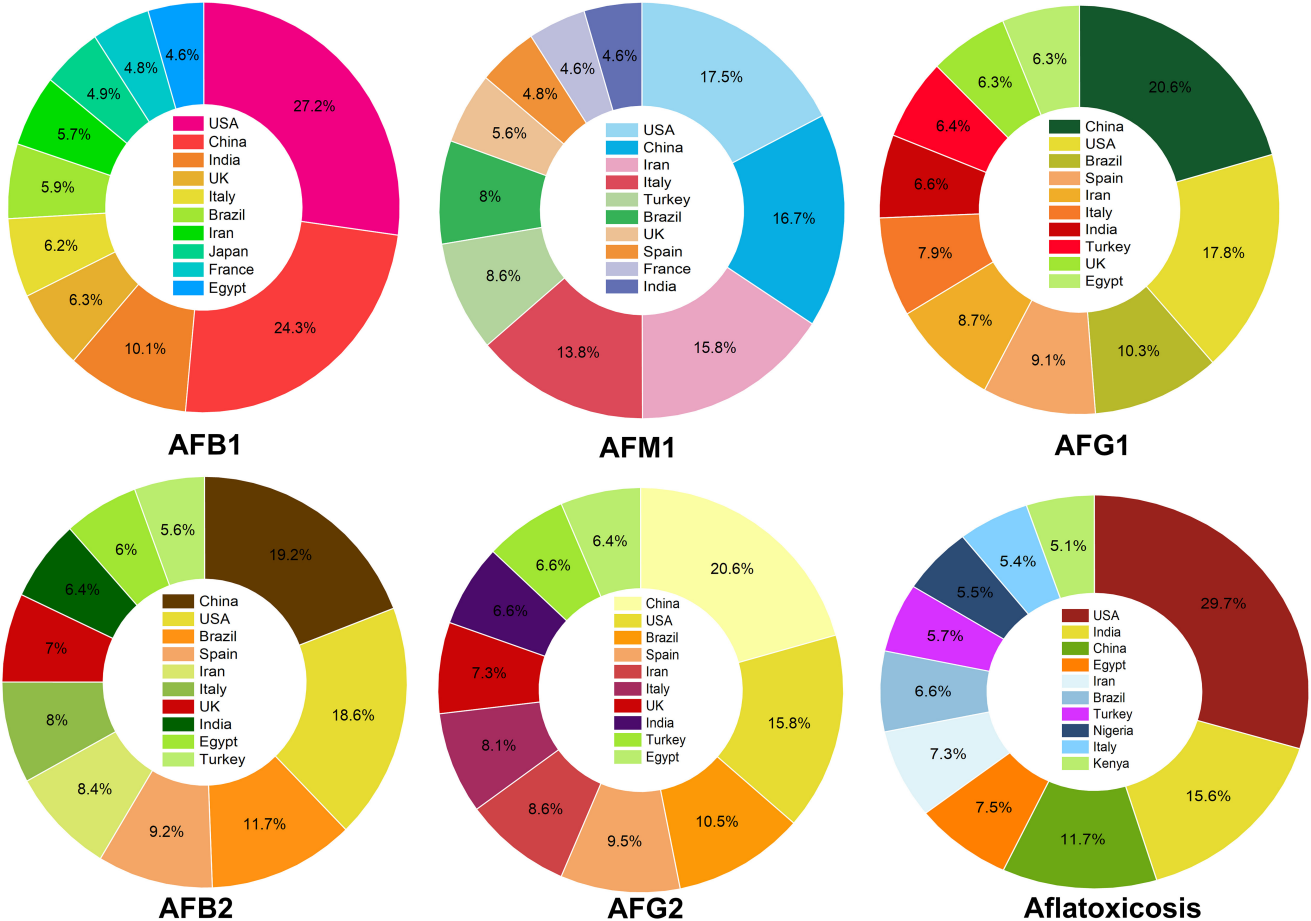
The proportion of academic investigations on AFs. For each class of AFs metabolites, the percentage of published studies has shown for the top 10 countries.

Evaluating the searched papers to highlight the top countries for research on AFs showed that the USA, China, and India were occupied the top ranks for studies on AFB1. In the case of AFM1, the USA, China and Iran were the three top countries to publish academic investigations on this aflatoxin. Indeed, China, the USA, and Brazil published many articles on AFG1 and ranked first to third countries to conduct research in this area. Interestingly, these results are in consistent with previous outcomes on the frequency of global studies on AFs in which the USA, China, and India were the top publishing sources from 1998 to 2017 (90). These outcomes together suggest that the global frequency of scientific studies on AFs has markedly extended over the past few years. Evaluating and monitoring academic publications on AFs can support researchers to identify the critical gaps in these studies for improving current regulatory policies, local and international awareness programs and making political decisions to protect target consumers from complications of AFs. Figure 4 shows a detailed representation on the proportion of the top 10 countries to conduct studies on different classes of AFs metabolites.

The review of literature unraveled that the conducted studies on AFs can be specified in different clusters. The majority of these studies targeted technical procedures for detecting and monitoring AFs in food sources, crops, spices, dairy products, nuts, and herbaceous products. Additionally, research interest in characterization of AFs in vegetable oils has been increased over the past 5 years. For example, a recent meta-analysis disclosed that vegetable oils such as sesame oil showed a differential AFs contamination (91). In another study, vegetable oil samples such as coconut oil was contaminated with different AFB1 concentrations (92). This indicates that consumption of such oil samples may pose serious health risks.

Analytical methods such as HPLC, ELISA, TLC, HPTLC, UHPLC, mass spectroscopy, and immunoassays were the most frequent procedures used for diagnosing the AFs contaminants in foods/feeds. Although the basic concept of these methods was previously reviewed in several studies (93–95), however, a fast and accurate method to characterize AFs in suspected sources has not been reported. Miklós et al. reviewed recent trends in developing accurate analytical/immunological measurements to identify AFs in different food and feed items (94). According to their finding, ELISA and LFIA are two promising methods to quantify the minimum concentrations of AFs in food or feed items. Indeed, IAC-clean up followed by HPLC-FLD is another accurate system for AFs measurements (94).

**Figure 5:**
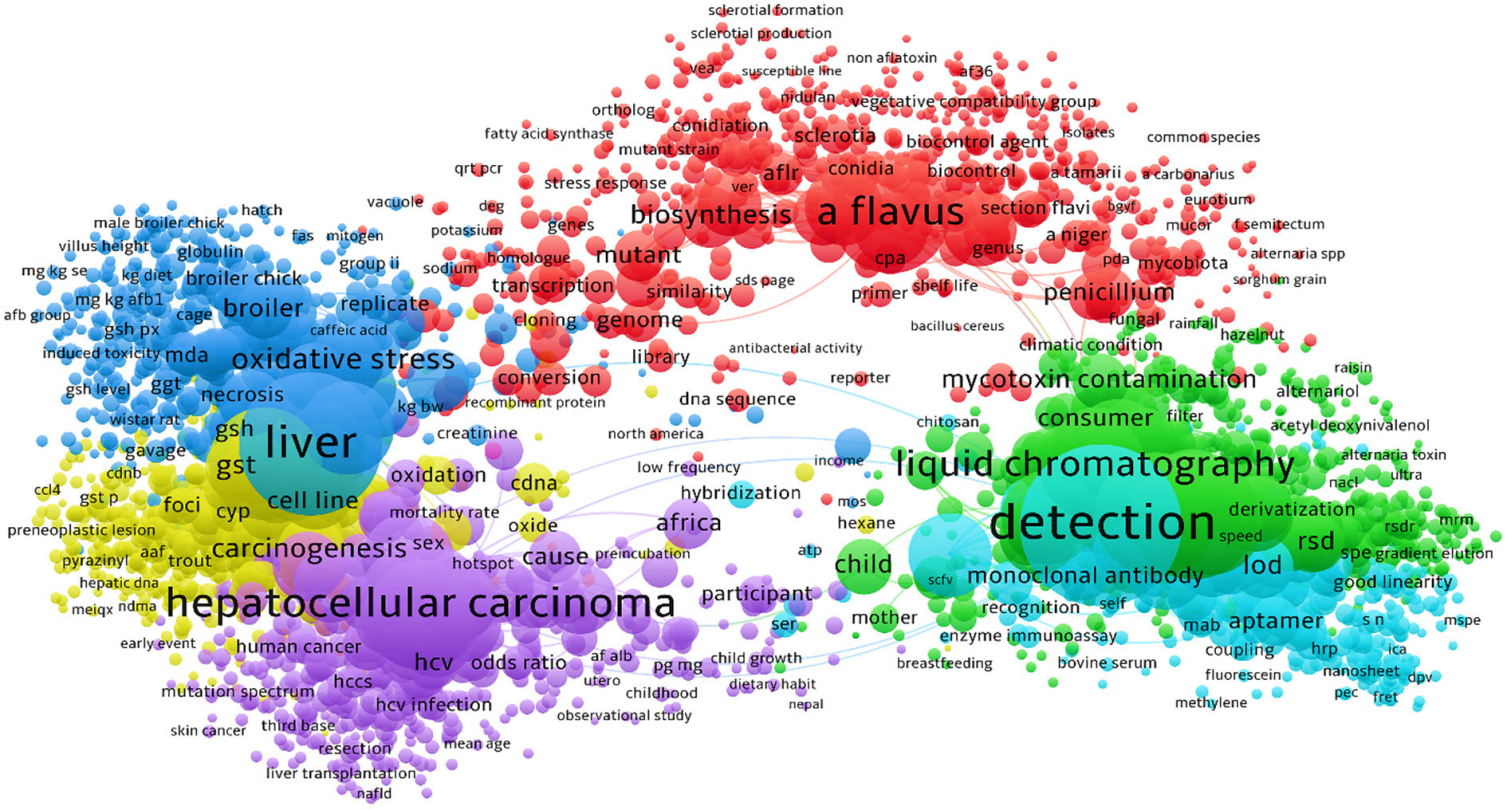
The bibliometric network of studies on AFs from 1990 to 2021 in scientific databases. As detailed in this figure, the majority of studies have been targeted the technical methods to identify the AFs contaminants in suspected samples. Research on carcinogenic effects of AFs was also occupied a large proportion of studies in this field.

Due to the co-occurrence of mycotoxins in food/feed items, the currently applied diagnostic methods might not appropriately detect different types of mycotoxins in evaluated items, therefore, the application of LC-MS/MS technique has been received much attention from academia owing to its ability in multiplex identification of AFs (94). As shown in Figure 5, a considerable number of studies also highlighted the carcinogenic effects of AFs and their role in the development of human cancers such as liver and gastrointestinal tumors. Studies on biological and chemical control of APF were also frequent among screened investigations.

We also found that studies on the effects of AFs on human and animal reproduction systems were increased during the past two decades. This is an interesting topic because the evidence suggests that AFs have negative impacts on reproductive organs (96). Studies on the toxicity of AFs for animals and chickens were also increased up to 5–10 folds over the past few years. More interestingly, the frequency of intervention studies using synthetic and/or natural compounds to alleviate the complications of AFs were also grown during the past decade. According to these statistical data, AFB1 and AFM1 were the most studied AFs during the past decades, though studies on AFB2, AFG1/2, and AFM2 were remarkably increased in the same time. Figure 5 highlights the major clusters for studies on AFs using searched keywords in scientific databases.

**Figure 6:**
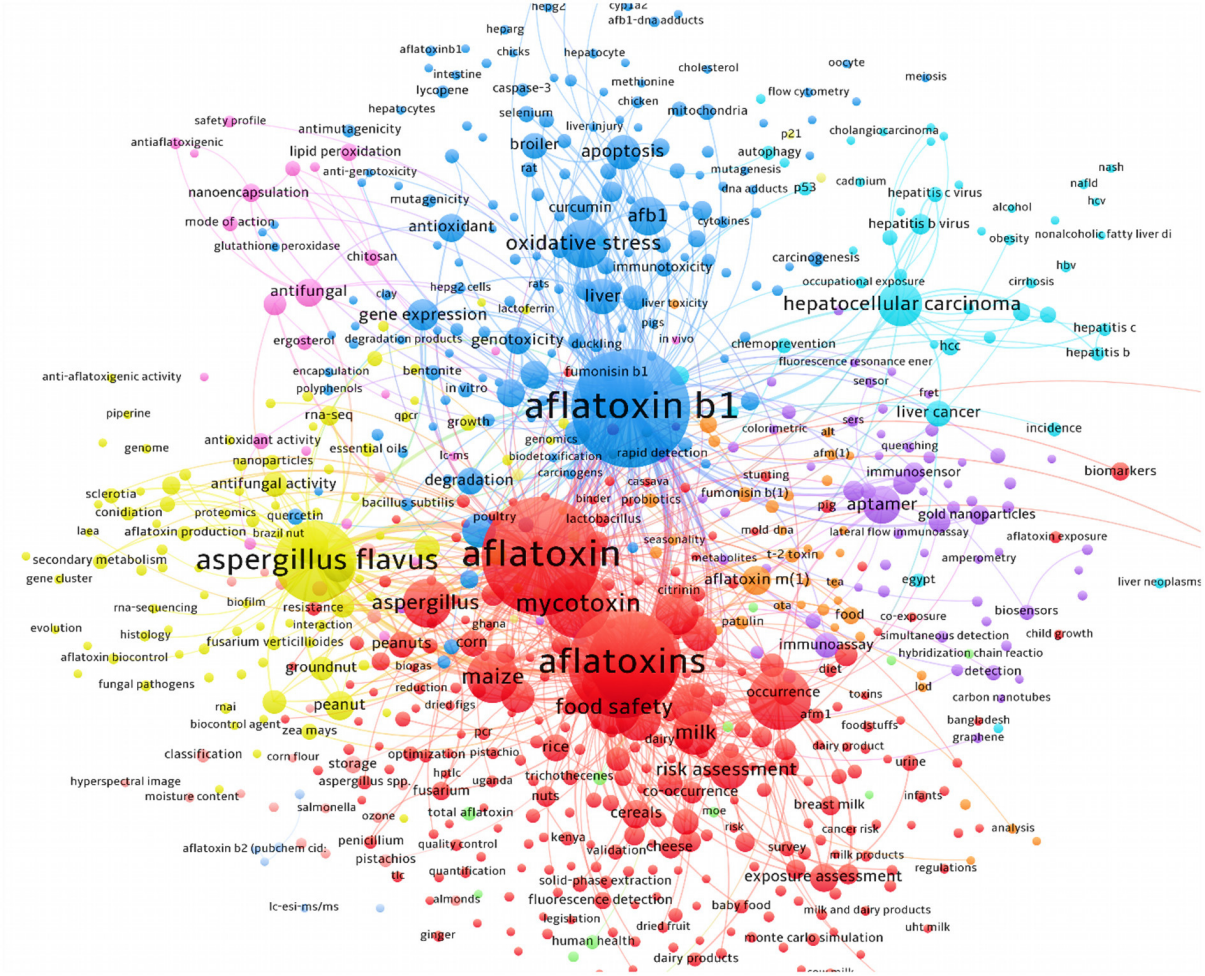
The detailed bibliometric network of studies on AFB1 in association with relevant fields.

Literature mining using available scientific resources has also manifested that the risk assessment investigations for characterization of AFs contaminants were dramatically increased. As depicted in Figure 6, the detailed bibliometric networks of studies on AFs unraveled that the carcinogenic effects of AFB1 in inducing hepatocellular carcinoma were significantly investigated. Interestingly, the outcomes showed that the application of PPs (e.g., curcumin and resveratrol) to alleviate health-related complications of AFs has increased during the last 10 years due to the health-promoting effects of these natural metabolites. In this regard, studies suggested that antioxidant therapy might ameliorate the hepatotoxicity of AFs metabolites, leading to lower health risks for cancer development (97). As it is discussed in the next sections, antioxidants are critical chemical agents provided the human body with ability to protect both lipid and protein elements from free radicals and oxidative agents (98). Therefore, considering antioxidants as promising agents in the prevention of AFs complications might decrease the deterioration of hepatic cells and prevent the development of liver cancer, though this claim requires further clinical assessment. Based on the data discussed in this section, it can be concluded that in which fields AFs have considerably studied and where they have been ruled out in scientific investigations. Because AFs are emerging health hazardous threats to the human society, studies on the complications of these fungal toxins, developing reliable medicines to decrease the toxicity of AFs and integrated management of AFs in production/storage sites should be increased to mitigate the quantity of AFs in food/feed items.

## Prevalence of mycotoxins in different geographic regions

**Figure 7:**
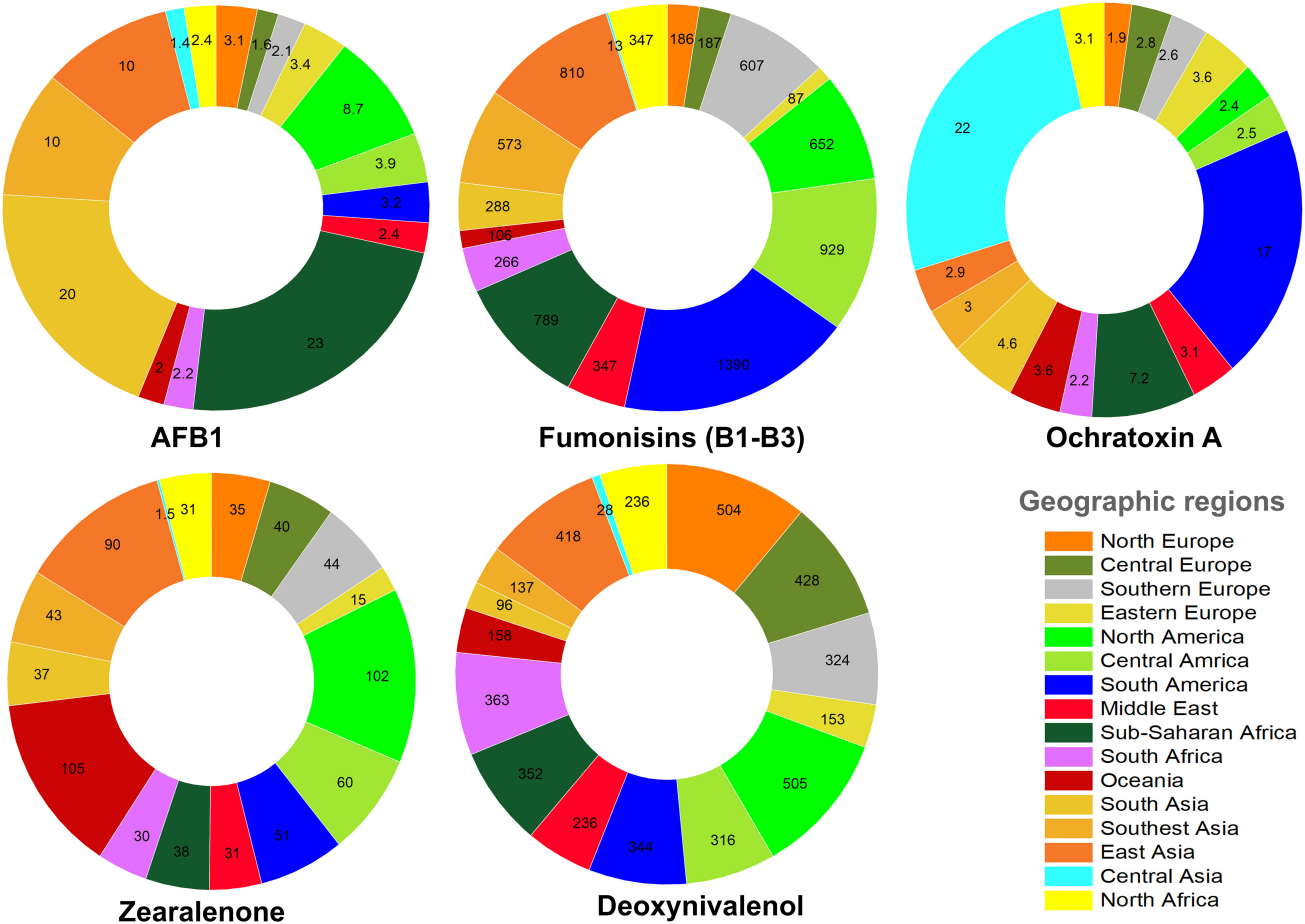
The median concentration (μg/kg) of AFB1 and other mycotoxins in feed from different geographic areas.

As discussed in the previous sections, the worldwide occurrence of AFs depends on several factors such as climate, the availability of standard crop/food storage facilities, the awareness of local farmers, food processing methods, post-harvest contaminations, and temperature and moisture of post-harvest storage sites. Of all, air humidity and temperature are two determinant factors to enhance the emergence of APF (99, 100). In an interesting study, the worldwide occurrence of several mycotoxins in feed has been summarized (101). Accordingly, in some geographic regions, the average concentrations of mycotoxin contaminants in feed are considerably higher than other ones owing to the imbalance distribution of these toxins. For instance, the highest median concentration of AFB1 was reported for Sub-Saharan Africa (23 μg/kg), South Asia (20 μg/kg), Southeast Asia (10 μg/kg), and East Asia (10 μg/k) (101). Fumonisins (B1, B2, and B3) are other mycotoxins that pose high health risks to the human societies. The highest median concentration of fumonisins was reported for South America (1390 μg/kg), Central America (929 μg/kg), and Sub-Saharan Africa (789 μg/kg) (101). Compared to other mycotoxins, fumonisins showed a broader geographic occurrence. In contrast, OTA displayed a domineering abundancy ratio in Central Asia (22 μg/kg) and South America (17 μg/kg), respectively (101). Figure 7 represents the worldwide median concentration of five mycotoxins found in feed in different geographical regions based on information adopted from Gruber-Dorninger et al. (101).

Other studies have also highlighted the observed level of AFs in different geographical regions (102, 103). The results showed that the occurrence of AFs in various foods, cereals, nuts, oilseeds and processed foods is inevitable and these sources showed a differential level of contaminations with AFs derivatives (102). According to these outcomes, the prevalence of AFs in each region might be affected by local climate and the abundancy of crops found in these regions (103, 104). Therefore, it can be concluded that AFs are almost not equally distributed in production/storage sites. Therefore, the construction of predictive models for the occurrence of AFs during specific seasons is helpful in identifying the contaminated food/feed items to prevent the circulation of AFs in local and international food chains (105, 106).

## AFs contaminants in cereals and nuts

Over the past decades, studies on various food/feed items to trace the fingerprint of AFs contaminants have been increased (13). Cereals are most commonly cultivated in the world, supporting human societies in reaching essential nutrients in their diet (10, 107). Studies reported that AFs occurrence in cereals is becoming a serious worldwide concern (11). The formation of AFs in cereals and cereals-based processed products depends on several factors such as fungal genotype, processing methods (drying, milling, blending, chemical additives), and environmental factors such as oxygen level, environmental pH, field temperature and humidity content (11).

A comprehensive analysis of different cereals samples using published records in GEMS/Food database showed that around 12.7% of all samples were positive for contamination with at least one of AFs (10). Correspondingly, rice, sorghum, and maize samples possessed a higher level of AFs (10). Other investigations also suggested that the highest level of AFs was detected in maize in the concentration of 3,760 μg/kg, which extremely exceeded from the USA and EU permissible standards (108). Among cereals, rice is surprisingly a susceptible crop prone to AFs pollutants (103, 104, 109). After rice, corn and sorghum are prone to AFs contaminants (109). The evidence suggests that AFB1 is the main AFs found in cereals (11).

Another study on 108 Brazilian wheat and wheat by-products samples disclosed that 30.6% of studied samples were positive for at least contamination with one of the AFs in which AFB1 was the most dominant fungal mycotoxin in these samples (110). The highest contamination levels were observed for wheat grains, followed by the barn, whole and refined flour (110). Presently in EU and other countries, only limited concentrations of AFs are allowed to be found in food products (36). In this regard, the allowed concentration of AFB1 and total AFs for nuts and cereals in the EU is 2 and 4 μg/kg, respectively (109). In cereals, applying inappropriate drying methods allowed for maintaining higher humidity content in these crops, leading to a higher ratio of AFs-contaminated crops (13). Recent studies on contaminated cereals demonstrated that differential concentrations of AFs are found in cereals grown in different countries (109). However, nuts, groundnuts, and cereals are prone to AFB1 contaminations under field and non-standard storage whenever temperature, humidity, and field soil are suitable for APF growth (13, 111).

A recent study on the prevalence of AFs in nuts from different origins demonstrated that peanuts from Argentina, Congo, Nigeria, and South-western Uganda were differentially contaminated with AFB1 (112). Accordingly, the average concentrations of AFB1 in peanuts from these countries were 530, 163.22, 110.95, and 103.10 μg/kg, respectively (112). Countries such as Taiwan, Morocco, and Iran ranked as first to third countries for contamination of pistachio with AFs (112). The maximum average concentration of AFB1 in almond, hazelnut, walnut, and Brazilian nut samples was observed for countries such as Cyprus, Italy, Morocco, and Brazil (112). Other interesting studies have also reviewed the contamination of Iranian pistachio with AFB1 using different procedures (113). Accordingly, the outcomes suggested that there has been differential AFB1 concentrations in this nut. Based on these results, around 37% of studies reported AFB1 contaminations in the concentration of ≥10 μg/kg, 35% of studies reported ≤ 10 μg/kg, and 28% of studies reported ≤ 5 μg/kg, respectively (113).

In another interesting study, Bui-Klimke et al. also analyzed the global regulations on prevalence of AFs in pistachio samples and reported that pistachio nuts are accounting for substantial quantity of dietary AFs (114). Accordingly, estimations showed that contaminated pistachio nuts remarkably affected the global market of this valuable nut and ignoring the presence of AFs in these samples will increase the health risks of these mycotoxins for target exported/imported locations (114, 115). Presently, global markets between Asia and other world countries have been spectacularly increased (116). Due to the higher rates of AFs occurrence in pistachio samples (114), top producers of this nut should develop modernized infrastructures for drying and processing pistachio to eliminate the expected level of AFs. Increasing monitoring gates in import/export gates of target consumers of pistachio can help to early detection of AFs sources and prevention of APF growth.

Walnut kernel and oil are two important products worldwide. Studies have shown that walnut kernel and oil have an interesting metabolic profile which in turn can be considered as a source of antioxidants and mineral elements (117, 118). Recent findings on the contamination of Iranian walnuts with AFs showed that nearly half of these samples were contaminated with AFB1 in concentrations of 0.8–14.5 μg/kg, respectively (119). Nuts such as walnut maintain remarkable moisture in their structure, leading to providing a favorable environment for APF growth (119). The grown fungi inside walnuts significantly affect these tree nuts quality and destroy their taste and flavor. Applying modern drying technologies to process, drying and shipping walnut and other nuts can extraordinarily decline the occurrence of AFs in such nuts. The prevalence of AFs in salt-roasted nuts is also becoming an emerging concern. In this regard, Ostadrahimi et al. (120) reported that salt-roasted pistachio and peanuts possessed a differential concentration of AFs. Correspondingly, the observed average AFs concentration in these samples was about 19.88 μg/kg in comparison to pure nuts (6.51 μg/kg), respectively (120). These outcomes indicate that processing of economic nuts should be carefully conducted because the occurrence of AFs after nut preparing steps for increasing their taste and flavor is inevitable; therefore, it can pose health risks to target consumers.

Considering the world climate zones map (Supplementary Figure S1), it can be said that the higher prevalence of AFB1 in peanuts harvested from different geographic regions may be related to the type and dominancy of countries' climate. The literature suggested that the moisture content above 17% and warmer temperatures (above 24°C) are effective in inducing the formation of AFs in corn and feed (121, 122). Indeed, the review of literature manifested that due to the higher moisture content of nuts, these products are the main susceptible foodstuffs for AFs contaminations (123). Therefore, in addition to local climatological factors, standard storage of nuts and decreasing the moisture content of these products before entering storage sites and local/global markets can dominantly affect the prevalence of AFB1 in such foodstuffs.

## AFs in animal products

A majority of AFB1/2 concentrations in contaminated crops have entered the animal feed network and metabolized to AFM1 (109). In this regard, the evidence suggests that nearly 1% of AFB1 metabolized into AFM1 in dairy cows (124). About 1–3% ingested AFB1 and metabolized AFM1 are excreted by feces and urine (109). However, the remained AFM1 level in the animal body will later enter the human food chain through dairy products. Due to the considerable affinity of AFM1 to dairy proteins (125), it seems that in dairy products with higher protein content (e.g., cheese), the occurrence of AFM1 is more probable compared to other AFs (109, 125). Generally, the occurrence of AFM1 in milk, cheeses, butter, and yogurt is surprisingly high; however, the final concentration of AFM1 in these products depends on processing methods and the quality of animal feed (109, 126).

Today, the presence of AFM1 is becoming a global concern (127), as discussed in previous sections, the frequency of studies on AFM1 has been dramatically increased over the past decades. A recent meta-analysis indicated that the prevalence of this mycotoxin among dairy products is averagely between 40 and 60% which has seriously been considered a biological threat to public health (128). Although the toxicity of AFM1, a hydroxylated derivative of AFB1, is relatively lower than other types of AFs (129), the available evidence indicates that the long-term exposure to AFM1 might be effective in the onset of liver cancer (39, 128). Animals supplemented with AFs-contaminated feed are major sources of AFM1. Because dairy products are unique sources of proteins, vitamins, and calcium, they are becoming the principal part of the human diet (127). Therefore, AFM1 contaminants in these products pose a threat to public health (128), and regular monitoring measurements should be conducted to diagnose target AFs in dairy products. However, to eliminate the AFM1 in animal products, increasing public awareness and regular monitoring of dairy products can be helpful (130).

Scientific studies also reported that the occurrence of AFs contaminants in livestock meat products is probable (131–133). Accordingly, AFs such as AFB1/B2 and AFG1/G2 with different concentrations occurred in meat-based foods (132–134). In an interesting study on meat products collected in Riyadh, Saudi Arabia, incredibly 37.5% of gathered samples were contaminated with AFs, and 4% of samples have exceeded from permissible standards (the acceptable Saudi limit: 20 μg/kg). Correspondingly, AFB1 and AFG1 were the most commonly identified AFs, followed by AFB2, respectively (133). In another study, the occurrence of AFs in meat products such as basterma, sausage, kofta, and luncheon was investigated, and the results unraveled that AFB1/2 occurred in higher concentrations compared to AFG1/G2 (132). Investigations on domestic fowls feeding diets containing AFs also displayed that the accumulation of AFs in their liver is higher than other organs (135). Indeed, the outcomes showed that the highest abundancy of AFB1 was observed for tissues of quails compared to other birds (135).

In another study conducted on meat, milk, and eggs samples, collected in Jordan, the outcomes indicated that the samples were contaminated with AFB1/2, AFG1/G2, and AFM1/AFM2 (136). In milk samples, however, the highest concentration of AFM1 was exceeded from EU standard for liquid milk (50 ng/L) (136). These outcomes suggested that the proportion of AFs contaminants in meat samples depends on several factors, including meat processing methods, post-storage contaminations, and non-standard transportation and shipment facilities of meat products to local and international markets.

Presently, the maximum permissible AFs concentration in animal feeds is 5 μg/kg based on EU limits (137). Therefore, preparing animal feeds from credited sources, improving of the storage condition of livestock inputs (137), improving the quality and accuracy of AFs detection systems (138), improving of animal feed manufacturing procedures (139), and regular monitoring of animal feeds to identify the source of AFs contaminants are possible strategies to reduce the concentration of AF pollutants in dairy products. Governments must seriously deal with providers of livestock feeds that might supply contaminated animal inputs to dairy farms by imposing strict limitations on their business to prevent further consequences of AFs. It is now well-documented that AFB1 was widely found in contaminated livestock products fed on contaminated forage and grain (140). Therefore, due to health threats of fungal toxins to public health, dairy products should be repeatedly monitored to decrease the quantity of AFs contaminants. Such strategies will later decrease the health consequences of AFs and help consumers to reach safe and AFs-free dairy items.

In practice, however, characterization of AFs-contaminated animal feeds is difficult because the available techniques require allocating sufficient time and financial support for early detection of different types of AFs metabolites (137–139). Governments should support researchers in developing accurate, fast and low-cost measuring systems to abate AFs metabolites from suspected feeding resources. As discussed in the previous sections, large quantities of scientific studies have been conducted on measuring processes of AFs diagnosing systems. Expanding academic studies without practical innovations cannot alone help the elimination of AFs in animal feeds; therefore, efforts to convert the results of academic investigations into touchable outcomes should be highly followed to recruit the power of science in controlling health hazardous mycotoxins. As we discussed later, AFs are major environmental risks factors in developing nCDs. Therefore, elimination of these fungal toxins in animal feeds can primarily decrease their occurrence in human food chain.

## AFs contaminants in spices and medicinal herbs

Spices are interesting food additives that constructed a valuable financial global market for spice-producing countries (141, 142). Presently, countries such as India, China, Nigeria, Indonesia, Thailand, Vietnam, Bangladesh, Nepal, Ethiopia, and Turkey occupy the first to tenth ranks of the top spice-producing countries (143). Estimations indicated that the demand for fresh, powdered, and processed spices have been increased over the past decades due to multi-functional applicability of these products for various purposes such as traditional medicine, cooking, etc. (142, 144). Interestingly, AFs contaminants also occur in spices with higher content of moisture. Since 2002, the EU has implemented rigorous regulatory policies to identify AFs in spices (36, 144). Accordingly, the permissible concentration of AFB1 and total AFs in spices has been reported up to 5 and 10 μg/kg, respectively (36). Studies proclaimed that well-distinguished spices such as pepper, ground red pepper, paprika, curcumin (or turmeric), chili, nutmeg, and ginger are the susceptible natural food additives prone to AFs pollutants (36, 109).

The evidence also suggests that the highest permissible AFs limits for all foods in the USA are 20 μg/kg (145). In Croatia, the permissible AFB1 and total AFs levels for spices are 30 and 15 μg/kg, while in Bulgaria, the accepted limits are in the concentrations of 2 and 5 μg/kg (144). Iran also follows the EU regulation on spices and the permitted maximum levels of AFB1, and total AFs range in the concentrations of 5 and 10 μg/kg (144). In this respect, several studies comprehensively reviewed the occurrence of AFs in spices (144, 146, 147). The outcomes of these scientific investigations demonstrated that the occurrence of AFs in spices depend on the type and processing methods of spices (144, 146, 147). Compared to cereals, and edible nuts, spices and plant-based food additives are highly prone to maintain higher humidity levels in their structure (148).

On such occasions, opportunist APF can easily grow among stored spices to decrease the quality, taste, fragrance, color and marketability of these popular plant-based food additives (149). In Asian countries, in particular Iran and India, food spices (or additives) are indispensable parts of daily cooking and different forms of spices including raw and processed stuffs can be purchased from local providers. Due to bulk production of spices, these products short immediately after harvest would send for local and international markets. Recent studies on herbal products and spices of different locations of Iran indicated that AFB1 is the most prevalent AFs among these spices and nearly 100% of analyzed red pepper samples were contaminated with AFs (148).

In another interesting study, different samples of commercial spices in Iran has been analyzed using HPLC method to identify the quantity and abundance of culprit AFs (149). According to these results, spices such as cinnamon, turmeric, black and red pepper diagnosed with different concentrations of AFs (149). Similar to the previous results (148), AFB1 was the most domineering AFs among analyzed spices, though different concentrations of AFB2, AFG1/2 were also observed among the evaluated samples (149). These results are in agreement with previous studies that confirmed herbal spices are remarkably prone to AFs contaminants (150, 151).

Monitoring of spices marketed in Africa also showed that the Ethiopian ground red pepper was extremely contaminated with AFB1 in a dose of 250–525 μg/kg (152). Simultaneous investigations on Iranian and Indian spices to detect AFs contaminants purported that spices from these origins were differentially contaminated with AFB1 in a concentration of 63.16–626.81 ng/kg (Iranian samples) and 31.15–245.94 ng/kg (Indian samples), respectively (153). More interestingly, the outcome of this investigation demonstrated that contamination of studied samples was not exceeded from EU standard concentration of AFB1 in spices (5 μg/kg) (153), though AFs metabolites were characterized in the monitored samples. In another study in Turkey, 93 spices and 37 medicinal herbs were evaluated to identify hazardous AFs derivatives. The results manifested that AFB1 was domineering fungal metabolite in nearly 32 herbs and 58 spice samples (154). Resultantly, the maximum concentration of AFB1 was found in cinnamon at the concentration of 53 μg/kg so that the measured concentrations in these samples were obviously exceeded from EU permissible values (154).

Evaluation of marketed spices in Doha, Qatar, showed that *Aspergillus* and *Penicillium* spp. were the most prevalent fungi in these samples (155). Interestingly, this investigation demonstrated that five spices, including turmeric, black paper, chili, tandoori and garam masala, were contaminated with AFB1 (155). For the first four samples, the detected AFB1 concentrations have deviated from EU standards (155). Indeed, according to the outcomes of a recent meta-analysis on the occurrence of AFB1 in red pepper, the prevalence of AFB1 among studied samples was 50.8%, respectively (156). This study reported that the minimum and maximum concentrations of AFB1 were detected in Korean and Turkey samples in the concentration of 0.14 and 31.13 mg/kg, respectively (156).

The growing body of evidence suggests health promoting medicinal plants are also prone to AFs contaminants because of their ability to support AFs-fungi growth (12, 157). In an interesting investigation the results displayed that AFB1 metabolite was found among medicinal plants with a significant prevalence percentage (12). The results also confirmed that other AFs metabolites, including AFB2 and AFG1/2, were also characterized in herbal supplies (12). These outcomes are in agreement with other studies that confirmed that AFs are commonly found among medicinal plants in different concentrations (157, 158). More interestingly, herbal products not only hosted AFs metabolites, but also various scientific reports confirmed that OTA is another hazardous mycotoxin found in these herbal stuffs (159). In some studies that revolved around the occurrence of AFs in medicinal plants, spices and herb-teas incongruous results come to view in which spices were contaminated with AFs while medicinal plant samples from tropical countries were free of these hazardous fungal metabolites (36). Evaluation of medicinal plants fungal flora also showed that *Aspergillus* species were the most superior fungal strains isolated from target medicinal herbs (160).

Monitoring of medicinal herbs in Thailand for AFs contaminants showed that these fungal toxins in the range of 1.7–14.3 ng/g have been occurred in these samples and AFB1 was abundantly detected in the evaluated herbal products (161). Although medicinal herbs are at risk of AFs pollutants, however, it is trustworthy to note that these herbal supplies have the ability to produce specific metabolites to detoxify AFs metabolites. In this regard, *in vitro* studies have shown that aqueous extracts of medicinal herbs such as *Centella asiatica, Hybanthus enneaspermus* and *Eclipta prostrata* displayed nearly 70% degradation of AFB1 (162). Meanwhile, it can be said that the concentration of characterized AFs in medicinal plants and herbal supplies depends on the type of plants, herbal processing methods, storage condition, variation of grown mycotoxigenic fungal strains, temperature and humidity of storage sites, secondary contaminations during herbal supplies storage, and the infestation of pests and plant pathogens to stored herbal products (163).

Contaminated herbal products in local markets are potential health risks to consumers because there are no accurate scrutinizing systems to monitor local herbal suppliers and identify contaminated commodities (164). The local and international markets of medicinal herbs are flourishing yearly, and it is now valued at more than 100 billion dollars (165). Therefore, by regular risk assessment of spices and herbal supplies as well as increasing the number of monitoring gates for local and international markets and also by increasing the quality of processed spices and herbal products and suitable storage and packaging of these supplies, official health and agricultural organizations can significantly mitigate the prevalence of AFs contaminations in this industry (144).

**Figure 8:**
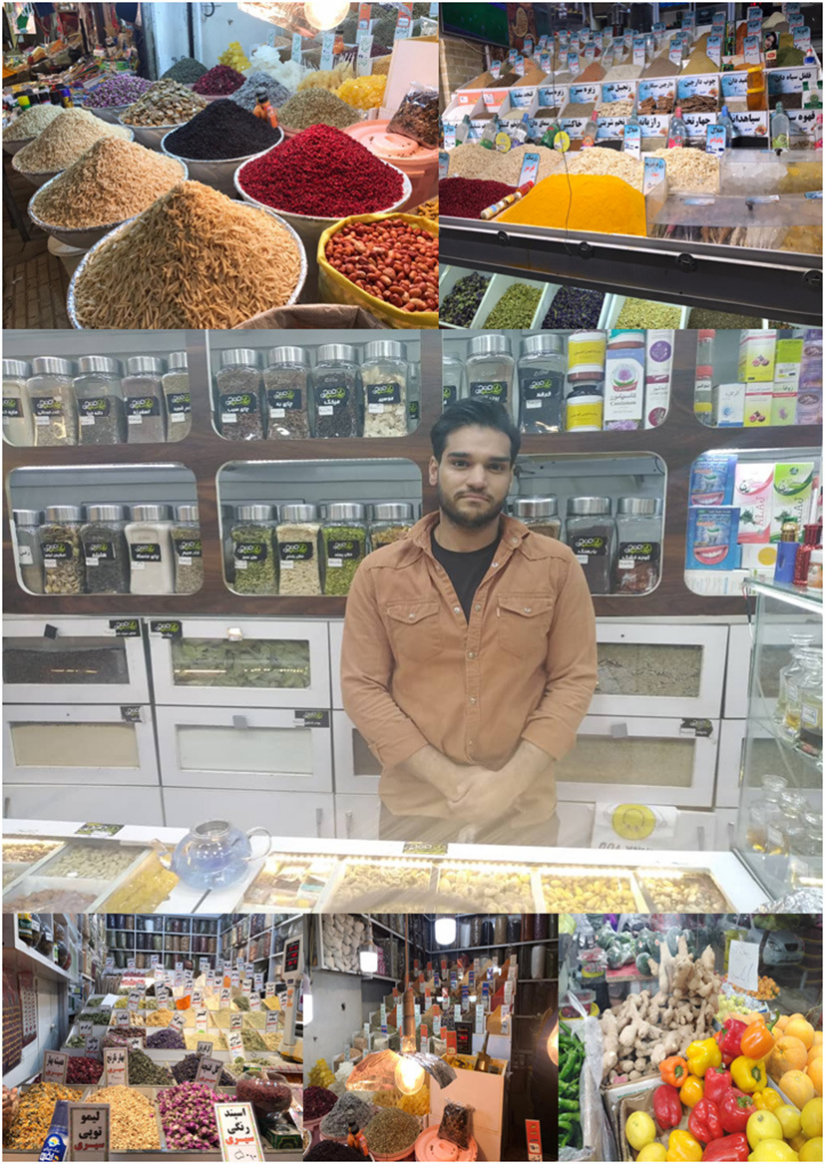
Local herbal and spices markets in Iran. Consumers should carefully check the flavor and taste of purchased spices and herbal products to reduce the health risks of AFs. The color of AF-contaminated spices, in some cases, changed significantly, and characterization of contaminations and spoiled materials is easy. In the case of invisible AFs contaminants, preparing regular samples from local herbal and spice markets will decrease the occurrence of these carcinogenic mycotoxins.

As shown in Figure 8, many herbal products and spices are marketed outdoors in local markets, which in turn might lead to post-harvest contaminations. Because herbal and spice providers might be not cautious enough to identify the source of AFs contaminants; sequentially, AFs-contaminated products may be intentionally or inadvertently passed to consumers, eventually leading to the progression of nCDs. Therefore, increasing the awareness of herbal farmers, manufacturers, and consumers is an effective strategy to eliminate AFs in such herbaceous products (166).

As discussed in previous sections, like many other foodstuffs, spices and herbal products were prone to AFs contaminants (148). In some cases, however, the occurrence of AFs is not in detectable concentrations. Additionally, due to the increasing demand for herbal spices and plant-based food additives (142), many national and international suppliers adhered to spice markets, leading to intensified cultivation and extensive processing of these products. The lack of fundamental infrastructures and suitability of climate factors enhanced the growth of APF, consequently leading to an increased level of detectable AFs contaminants. Therefore, to mitigate the total level of AFs in spices, medicinal herbs, and other popular plant-based food additives, local and international herbal markets, however, should be constantly monitored for detection of AFs metabolites (167, 168). Keeping in mind the most popular quote perhaps assigned to Hippocrates (400 BC) “*let food be thy medicine and medicine be thy food”* (169), healthy foods are indispensable parts of our dietetic regime and are complement to modern pharmacology (169). Therefore, preparing foodstuffs from mycotoxin-free sources not only improve our lifestyle, but also can decrease the progression of nCDs and improve the quality of daily diet.

In this regard, to bring concentration of AFs in spices and herbal products down, taking precautionary actions, such as raising public awareness, might support consumers to purchase healthy and AFs-free products (170, 171). According to scientific data, AFs contaminants in herbal products used in traditional medicine mainly occur in two stages during drying/processing and storage of target herbs/spices (172–174). Therefore, the lack of strict regulations on herbal ingredients used in traditional medicine might increase the occurrence of AFs in these health-promoting products (174). It is pivotal to implement rigorous regulations on the production, processing, packaging, manufacturing and exporting/importing of herbal products that are prone to AFs contaminations. Improving packaging systems (108) and developing standard infrastructures for the distribution, storage, and transportation of medicinal herbs and spices can also help consumers to use more safer products (175, 176). Providing safety guidelines for preparing herbal products and spices helps the public to broaden their knowledge about the consequences of AFs and associated health complications; therefore, leading to increasing demands for mycotoxin-free commodities and a healthy lifestyle. In the next sections, we will discuss the circulation of AFs in the human body and major AFs health consequences reported in the literature. This helps readers to a better understanding of AFs biological properties and their role in developing human chronic diseases.

## Interactions of AFs and human serum albumin protein

Studies have shown that AFs metabolites are prone to bind human serum albumin (HSA) (177). HSA is one of the most prevalent proteins in human blood plasma (178). HSA is a globular protein produced in the liver and constructed from a monomeric structure with several subdomains (179, 180). HSA functions as a carrier in the human body to transport fatty acids, drugs, hormones, and other biomolecules (178). This multifunctional, negatively charged, and non-glycosylated protein also participates in the regulation of plasma osmotic pressure. HSA is formed from 585 amino acids, and its 3D crystallographic structure is well-documented (179).

Structurally, HSA spatial conformation is formed by a heart-shape molecule, possesses three helical domains (I, II, and III), and is divided into A and B subdomains (IA, IIA, IIIA and IB, IIB, and IIIB) (178, 179, 181) (Figure 10). In the structure of HSA, there are two distinct binding sites, including Sudlow's I and II, each prone to bind different types of chemical agents (178, 182). Generally, negatively charged large heterocycles bind to site I, whereas small molecules prefer to interact with site II (182). The decreased concentration of HSA in blood plasma is associated with AD (183), cancer, obesity, diabetes, heart failure, stroke, and venous thromboembolism (178, 184).

**Figure 9:**
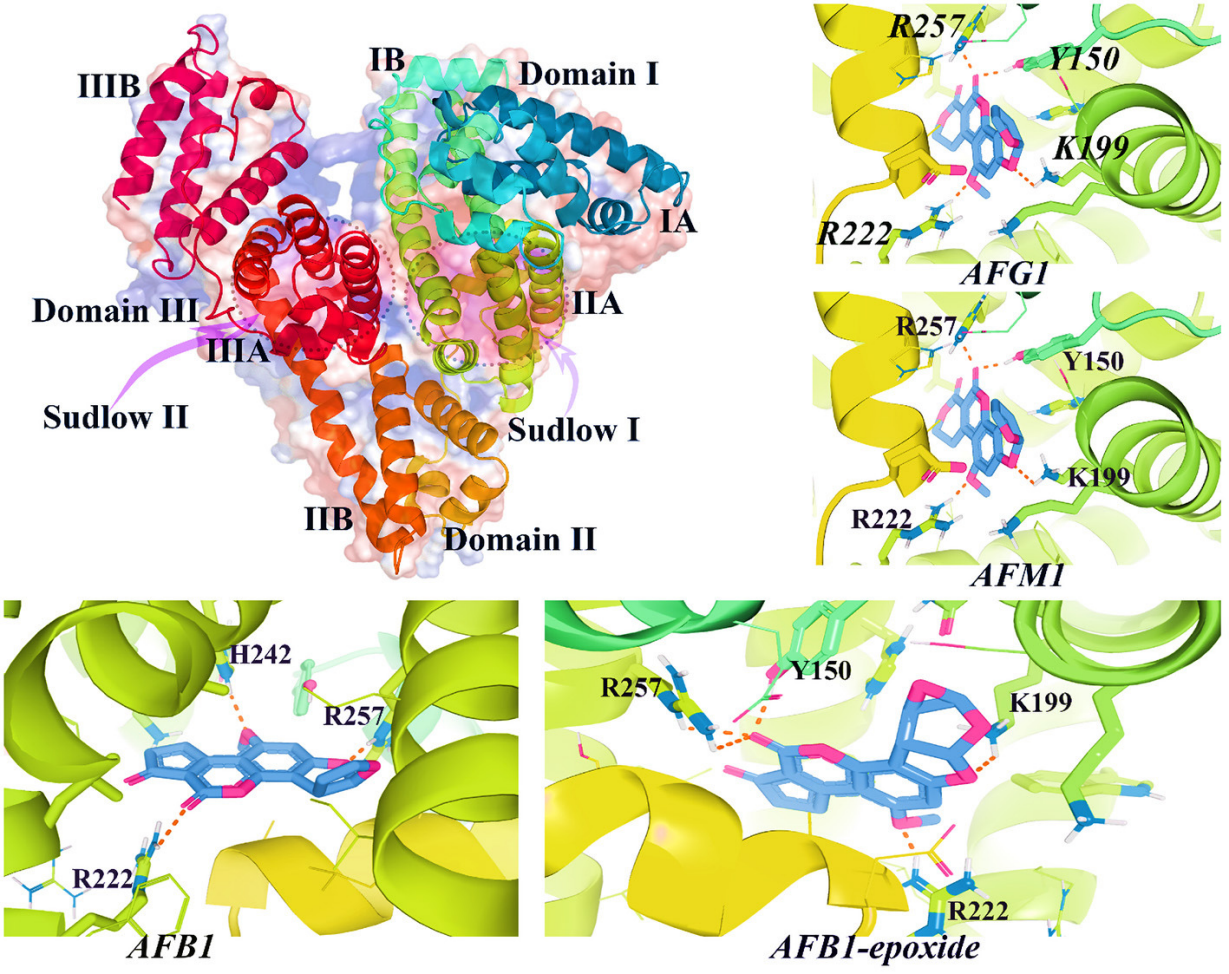
The 3D structure of HAS (PDB id: 1AO6) and interacted AFs metabolites with HSA binding site I. As shown in this figure, AFs metabolites bind to critical active sites of HSA by forming H-bonds and other relevant chemical bonds.

HSA plays a critical role in the tissue distribution of AFs metabolites (185). To date, only few studies have been conducted on the possible interaction of AFs and HSA binding sites. The available evidence suggests that AFs could non-covalently bind to HSA binding cavities (185). Evaluating the binding mode of chemical ligands to target receptors helps researchers to characterize the molecular behavior of these molecules *in vivo* (186). To understand how AFs metabolites might interact with HSA, we computationally investigated the binding affinity of AFs and AF-derived metabolites and HSA binding sites. As explained, only few studies are available to show the exact binding mode of well-known AFs to HAS (185). Therefore, to broaden the literature consistency on this topic, as part of this review 14 AFs metabolites, including AFB1/2, AFG1/2, AFM1/2, AFB2a/G2a, AFP1, AFH1, aflatoxicol, AFB1exo-8,9-Epoxide-GSH, AFB1exo-8,9-Epoxide, and aflatoxin-N7-guanine have been structurally prepared and docked into HSA using supervised and blind docking protocols (187, 188). The docking results for the interaction of AFs metabolites and HSA binding site I showed that docked AFs possessed different binding affinities to interact with HSA site I residues. The calculated binding energies for these metabolites ranged from −6.2 to −9.5 kcal·mol^−1^, respectively (Figure 9). AFB1exo-8,9-Epoxide, AFB1, aflatoxicol, AFG1/2, and AFM1 significantly formed H-bonds and Van der Waals forces to interact with HSA binding site I.

Interestingly, docking results demonstrated that AFs metabolites such as aflatoxicol, AFB1, and AFB1-exo-8,9-epoxide, might also interact with HSA subdomain IB. Previous studies suggested that AFB1/2, AFG1, and AFM1 mainly interacted with HSA binding site I (185), but the binding affinities of the remaining AFs metabolites are not comprehensively investigated in the literature. In the case of other mycotoxins such as zearalenone, the binding mode inhibitory assays showed that this fungal toxin could strongly bind to a non-conventional binding cavity between Sudlow's site I and II (189). More interestingly, OTA has two binding sites in the structure of HSA with different binding constants so that the highest binding affinity for this toxin was observed for subdomain IIA HSA protein (190). Other interesting experimental studies also confirmed that AFB1 is mainly bound to HSA in binding site I located in subdomain IIA with a binding affinity around 10^4^ M^−1^ (177, 191). Similarly, the results of spectroscopic and computational assays also determined that AFB1 and AFG1 also interacted with subdomain IB residues in HSA (192). AFB1 also displayed a similar binding affinity to interact with bovine serum albumin (BSA) binding site I with a binding constant of nearly 4.20 × 10^4^ M^−1^ (193). These outcomes together demonstrated the precise interaction of AFs and HSA, leading to a better understanding of toxicokinetic properties of these mycotoxins. Therefore, displacement of HSA-AFs complexes has been suggested as a therapeutic strategy to diminish the affinity of these mycotoxins to HSA and decrease the tissue delivery and uptake of AFs (177).

Decreasing the affinity of HSA to AFs with chemical compounds sharing similar binding patterns with higher affinities in comparison to AFB1 might bring down the toxicity of this mycotoxin for the human body (193). Studies have shown that natural products such as PPs interfere with the interaction between AFB1 and HSA and reduce the transportation of AFs to delivery locations (177, 194). More interestingly, scientific outcomes reported that administration of vitamins A and E could reduce carcinogenic properties of AFs in studied animals (195–197), though controversial results on the protective roles of vitamin E in cancer therapies have been reported (198). These findings are in agreement with previous outcomes demonstrated that exposure to AFs metabolites is associated with plasma micronutrient deficiencies (199). However, mycotoxin metabolites could bind to HSA (189, 193); therefore, these toxins are easily transported to different parts of the human body and causing chronic health consequences (189). In the next section, we explain how metabolized AFs derivatives are accounting for prevalence of nCDs. The discussed nCDs have been selected based on the frequency of conducted studies on each field of interest.

## May AFs promote the onset of diabetes mellitus?

DM is a chronic metabolic disease mainly characterized by elevated blood glucose level and insulin deficiency (200, 201). More than 422 million people are expected to suffer from DM in which the number of DM affected people in low-income countries has steadily grown during the past decades (202). To date, various subtypes of DM have been identified by which scientists can treat affected individuals through observed symptoms. Generally, type 1 DM (T1D) and type 2 DM (T2D) are the two the most prevalent subtypes of DM, leading to thousands of deaths yearly. T2D is responsible for more than 95% of all diabetic cases, while T1D only represents 5% of diabetic individuals. T1D is more prevalent among juvenile people and is significantly dependent on insulin deficiency (188, 203). In contrast, T2D is insulin-free DM, by which affected people suffer from elevated blood glucose and associated complications (203).

The emergence of modern drug design technologies leads to the development of potent anti-diabetic drugs. Different anti-diabetic medicines with specific molecular targets have presently been introduced into global markets (200). However, these drugs could not entirely suppress the complications of DM (200, 201), in turn, leading to an increased economic healthcare cost that allocated on caring for DM-affected people.

Recently, the role of exposome measurements has been highlighted in the progression of DM (204, 205). Exposome-associated factors can be divided into external and internal factors. External factors are features that directly linked to nearby environment such as pollutants, chemical materials, lifestyle and dietary regimes (205). Instead, internal factors are accounting for epigenetics alterations, gut microbiota and relevant molecular processes (204). This ongoing paradigm helps to understand how and where exposure to environmental factors lead to the progression of MetSys and other human diseases (204).

As a complicated metabolic disorder, DM progression depends on various factors (206–208). By considering exposome-associated factors in the development of DM, it is worthy to note that the adopting of a healthy lifestyle can decrease the incidence of this metabolic disorder (206). Environmental factors such as exposure to hazardous chemical agents (209) and toxins might increase the onset of DM (210). Indeed, the complex interaction between environmental and genetic risk factors might worsen the health complications of DM (206, 211). Biological toxins might act as health hazardous diabetogenic agents to disrupt normal function of the human body in controlling blood sugar levels and associated signaling pathways (212).

In this regard, evidence-based studies imparted that long-term exposure to particular types of AFs, such as AFM1, might increase health risk factors for developing T2D and other metabolic disorders (213). Interestingly, long-term exposure to AFB1 increased liver injuries in mice, disrupting blood glucose levels, insulin sensitivity, and a high chance of inducing liver cancer (39). Recent studies have shown that type 1 diabetic mice exposed to AFB1 showed a significant reduction in MUP1 levels, in turn, indicated an elevated blood glucose level and decreased insulin sensitivity (214). Molecular mechanisms underlying the *diabetogenic effects* of AFs are not completely understood, however recent investigations reported that AFs metabolites, in particular AFB1, might influence the regulatory switches of specific signaling pathways, genes, transcription factors, and receptors such as IGF2 and IGF1 receptor IGF-IR (215). In this regard, the evidence suggests that the increased level of IGF2 expression in pancreatic islets is associated with the onset of DM and dysfunction of β-cells (216).

The overexpression of IGF2 affects the functionality of β-cells, leading to chronic endoplasmic reticulum stress and dysfunction of pancreatic islets (216). Indeed, the evidence also suggests that IGF1 plays a critical role in DM by lowering blood glucose levels and insulin secretion (217). Therefore, the interaction of AFs with such molecular targets might negatively cause molecular abnormalities, which later lead to the development of DM. Additionally, hepatorenal injuries, lipid peroxidation, DNA damage, oxidative stress, and inflammation are other symptoms of animal models exposed to AFB1 metabolite (218).

In an interesting study (219), it has shown that the long-term exposure to mycotoxins was significantly associated with DM development in affected rats (219). In this finding, OTA could remarkably increase blood glucose levels, cause damage to pancreatic islets, and decrease insulin secretion (219). The cross-talks between the progression of MetSys and prevalence of HCC have been widely investigated (220, 221). The evidence suggests that MetSys might be connected to the progression of cancer (221). Yesheng et al. meta-analysis (221) reported a possible link between MetSys and pathogenesis of HCC among Euro-US societies, though there has not been association between HCC and MetSys clinicopathological feature (221).

In another study, Marchioro et al. (222) reported that in broilers chickens supplemented with a mixture of AFs (B1/2-G1/2) in the concentrations of 0.7–2.8 mg/kg for 42 days, chickens' performance features and enzymatic activity of pancreas have notably been altered (222). The outcomes imparted that long-term chronic exposure to AFs mixture increased the activity of pancreatic α-amylase and lipase while trypsin levels has been affected by the maximum concentration of AFs mixture (2.8 mg/kg) (222). The literature has disclosed that AFs altered the accumulation of lipids droplets and lipoproteins in addition to the dysregulation of lipid metabolism-related genes (*CHO, TAG, PHOL, MDA, Lipc, Lcat, Scarb1*, etc.) (223, 224). The evidence imparted that the dysregulation of fatty acids, cholesterol, and other health affecting lipids biosynthesis and metabolism is accounted for the progression of DM (225). Therefore, exposure to AFs in the dose-dependent fashion might contribute to the development of DM and cardiovascular diseases *via* alteration in the body lipids metabolism pathways (223, 226), though this claim requires future confirmation.

In rats exposed to penitrem A, a highly toxic mycotoxin from *Aspergillus* genus, a considerable diabetogenic properties has been observed (227). In this regard, chronic exposure to OTA (45 μg/daily diet) for 6–24 weeks caused a significant decrease in insulin levels and increase in blood glucose and glucagon levels (227). The observed diabetogenic activity of OTA is attributed to its impact on degeneration of pancreatic Langerhans islets (227). The elevated diabetogenic effect of mycotoxins in combination with chemical agents such as insecticides has also been investigated (228). Correspondingly, the outcomes displayed a remarkable synergistic interaction between mycotoxins and chemical agents in the onset of DM by increasing blood glucose and dysregulation of liver enzymes (228).

In a cross-sectional study conducted on Guatemalan participants, the outcomes manifested a significant association between AFB1-albumin adduct levels and pathogenesis of DM (229). Additionally, there was no significant association between AFB1-adducts and the progression of other metabolic diseases such as central obesity, obesity, non-alcoholic fatty liver diseases (229). This result, however, was aligning with previous animal-based studies that confirmed the association between exposure to mycotoxins and the onset/progression of DM (219). The evidence imparts that fungal toxins may increase the susceptibility to the onset of MetSys; therefore, well-designed human-based studies are needed to show how mycotoxins and AFs may contribute to the progression of MetSys (213, 230).

## AFs and pathogenesis of Alzheimer's disease

According to WHO statistics (231), there are more than 50 million AD-affected people worldwide such the statistics that have projected to increase by 2050 (187, 232). This prevailing neurodegenerative disorder is chiefly characterized by a remarkable decline in thinking, memorial dysfunction, unpredictable behaviors, language problems, and cognitive impairments, in turn, sequentially causes significant damage to the brain cells (231–233). Accordingly, the lesion of brain cells, the accumulation of amyloid plaques, neurofibrillary tangles, oxidative stress, NIF, and synaptic dysfunction are typical clinical symptoms of AD (187, 232, 234).

Different hypotheses have been postulated for the progression of AD; however, it is not completely clear which of molecular switches drives the inception of AD to cause obvious damages to the brain (234, 235). Scientists suggested that environmental and genetic risk factors, exposure to chemical pollutants, heavy metals, mycotoxins, lifestyle, age, infections, cardiovascular dysfunctions, T2D, cellular senescence, and head injuries may play a critical role in pathogenesis of AD (232, 236–238). Studies have shown that the AFs metabolites can alter various brain enzymatic actions, leading to AD development. For instance, in rodent models, exposure to AFB1 could significantly decrease the activity of brain protein kinases (239). The SH-SY5Y human neuroblastoma cell lines exposed to 100 and 50 μM AFB1 and FB1 mycotoxins for 24 h, manifested a significant increase in ROS formation, though the trace of endoplasmic reticulum stress was not observed (240). On the contrary, in adult male rats treated with 25 μg/kg/week AFB1 for 8 weeks, AFB1 could trigger obvious neurotoxicity, inflammatory responses, oxidative stress and, anxiety and depression-like behaviors (241). The finding showed that AFB1 supplementation was linked to a reduction in the activity of GSH, GST, SOD, and GSH-Px enzymes and increased MDA, IL-1 and TNF-α levels in right region of cerebral tissues (241).

The AFB1 also negatively influenced the distribution of astrocytes in rats' cerebral cortex and hippocampus (242). The effects of AFs on the BBB were also investigated such that the outcomes showed that AFs (in particular AFB1) could alter mitochondrial gene expression profile in the human BBB cells model (243). More interestingly, AFB1 could inhibit the electron transport chain function, affect ATP synthesis and dysregulate key genes in mitochondria (243), leading to genetic mutations and DNA damage (244).

The AFB1-NIF is attributed to the interaction of AFB1 metabolized derivatives with neuroinflammatory signaling pathways (245). It is now well-established that neuroinflammation promotes the pathogenesis of AD and other neurodegenerative diseases (246). The molecular mechanisms underlying neuroinflammation have partially been investigated, however, little is known on how exposure to mycotoxins may have impact on the incidence of AFB1-NIF (247).

To elucidate the AFB1-NIF mechanism of action, it is important to take this question into consideration how AFB1 metabolites may alter the NIF signaling pathways? Briefly, the activation of microglial cells elevates glial neuroimmune responses (248, 249). Next, CNS-related genes might be up- and/or down-regulated, sequentially resulted in the reactivity of astrocytes and expression of pro-inflammatory molecules such as IL-1/1β/6, INF-γ, and TNF-α (248). Activation of these neuroinflammatory signaling mediators will increase the ROS/RNS levels in the brain, leading to a significant oxidative/nitrosative stress and neuronal damage (246, 250).

Studies have proven that TLRs, MAPK, MyD88, CxCR4, PI3K/AKT, mTOR, COX-2, iNOS, Nrf2, HO-1, γ-enolase, STAT, AMPK, JAK, and NF-κB signaling pathways are major components of NIF (248, 251, 252). The alteration of kynurenine/tryptophan ratio (253), dysregulation of intracellular protein kinases (PKs) (254), the loss of neuronal integrity (255), and dysregulation of neurotransmitters signaling circuits are other pivotal components of NIF in response to brain abnormalities (256). The evidence introduced thus far supports the scenario that AFB1 enhances the secretion of pro-inflammatory cytokines such as TNF-α and IL-6 in CNS-derived cells, leading to the promotion of immune responses and significant oxidative stress in the CNS (247). These elevated level of neuroimmune reactions and activated signaling pathways in astrocytes and glial cells have been reported as consequences of AFs (257). Interestingly, low and high-dose exposure to AFs might alter the activity of brain signaling cascades based on exposure time duration and toxicodynamic properties of culprit AFs (258).

The acute exposure to AFs could notably affect the expression of genes and enzymatic activation in the brain. In rats, acute treatment with AFB1 influenced the activation of protein kinase C by phosphorylation of Ser957 position in the cerebral cortex (259). CCK is another critical protein kinase in the brain accounted for pathogenesis of AD (260). Studies have revealed that the functionality of brain cells depends on ATP molecules produced by CCK (245). Blocking CCK activity is associated with energy depletion in the brain, which can lead to significant oxidative stress and brain abnormalities. AFB1 inhibited the CCK enzyme to decrease ATP metabolism and trigger oxidative stress in the brain (245, 261).

Park et al. reported that AFB1 decreased human astrocyte cell proliferation by arresting cell cycle, sequentially induced the mitochondrial dysfunction and apoptosis of astrocytes (262). Accordingly, the interesting part of this finding that is AFB1 dysregulated calcium hemostasis and increased ROS formation, leading to neurotoxic effects on astrocytes cells *in vitro* and *in vivo* (262). In female Wistar rats given 15.75 μg/kg/orally for 8 weeks, the outcomes suggested that AFB1 decreased the distribution of astrocytes in frontal cortex without effect on neuronal numbers (242). In contrast, AFB1 increased neuronal number and decreased astrocyte distribution percentage in the hippocampal CA1 subfield. Importantly, the withdrawal of AFB1 restored the observed changes in rat brain (242).

In the support of these outcomes, Alsayyah et al. reported that the severity of chronic neurodegenerative effects of exposure to AFB1 is associated with astrocyte immune responses and alteration of brain enzymes (239). Accordingly, chronic exposure to AFB1 altered the activity of antioxidant enzymes (GPX, CAT, SOD, GSH), increased the activity of AP and LDH, and decreased CK activity (239). The observed AP and LDH increased activity are attributed to neuronal death, astrocytes damage, and necrosis (239). This finding also indicated that the chronic side effect of long-term exposure to AFB1 depends on the passed quantity of these toxin from the BBB and duration of exposure (239). In another study, animals feed with 5 ml AFB1 for 8 weeks also showed noxious neuronal degenerative changes in cerebral cortex (263). Another animal-based investigation also manifested that AFB1 increased the activity of AChE and ADA enzymes (264). The up-regulation of these enzymes might be responsible for elevated level of inflammatory responses due to tissue damage. Additionally, AChE and ADA may be contributed to clinical signs of apathy because of their participation in neuromodulation and neurotransmission (264).

By the way of illustration, the evidence suggests that AFB1 triggered acute neurodegenerative consequences in the CNS, induced encephalopathy by influencing glutamate neurotransmitters, increased ATP depletion, modified brain catalase, SOD, MDA levels, and GST concentrations (245). What is outstanding in these outcomes is that differential exposure to AFB1 induces oxidative stress (265, 266) and neuronal damage in the brain (245). Additionally, exposure to AFB1 is associated with reduction in CNS phagocytic ability, increased levels nitrosative stress, increased expression of cytokines (TNF-α and IL-1β/6/8/10) (267), induced microglia cell apoptosis, dysregulation of *p*-NF-κB signaling pathway (268), significant alteration in brain integrity, substantial DNA damage, S-phase cell cycle arrest (269), and other neuroimmunotoxic complications (245). AFM1 also could degrade the BBB structure by influencing astrocytes, vascular endothelial and microglia cells to trigger remarkable neurotoxicity in the brain (262, 270). These data indicated that AFB1-NIF affected brain enzymatic and none-enzymatic reactions as well as other CNS molecular components, ultimately leading to the onset of AD.

As an epidemiological standpoint, the clinical framework of AD pathogenesis and exposure to AFs has still not transparent, consequently further well-supervised trials should be conducted to know how do exactly these mycotoxins contributed to molecular dysfunctionalities in the brain (245). As explained earlier, the neurotoxicity and side effects of AFs in the brain have been documented through *in vitro* and animal studies, therefore, for cautionary reasons the elimination of these mycotoxins in food/feed should be repeatedly followed to reduce their clinical end effects.

## AFs and the onset of cancer

Cancer is one of the most lethal chronic diseases, leading to millions of deaths yearly (271, 272). According to global statistics (271, 273), the prevalence of cancers is remarkably increased during the past decades (271, 273). Still, an efficient and safe medicine for the treatment of cancer has not been introduced (274). Among all influencing environmental and genetic risk factors, the carcinogenic effects of fungal toxins in contaminated foods raised global concerns about the prevalence of cancer (271). As discussed, studies have reported that AFs are toxic, carcinogenic substances (154). These fungal metabolites disrupt the normal activity of signaling pathways, gene expression, and enzymatic activities in the human body. The evidence suggests that long-term exposure to high concentrations of AFs remarkably influences liver and kidney function (19). Briefly, AFs affect the expression level of many genes involved in phase I and II metabolism in the human body (275). Compared to other xenobiotics, the highly liposoluble AFs are rapidly absorbed at the site of exposure (276). Studies have shown that respiratory tracts and gastrointestinal organs are two major sites for the absorption of AFs into the body (276, 277).

AFs affinity to carrier proteins in the body helps these carcinogens to enter the blood and circulate around tissues and organs (191, 194). Highly toxic and reactive AFs metabolites bind to DNA to form AFs-DNA adducts (278, 279). Studies have reported that the interaction of AFs and DNA causes significant damage to DNA and associated biological processes such as transcription and chromatin packaging (280). Binding AFs to DNA and key enzymes in the liver and other organs can induce cancer in different ways. Studies suggest that occurring mutation in specific sites of DNA and proteins is linked to the pathogenesis of cancer (281).

The formation of AFs-DNA adducts can affect the topology of packaged DNA and DNA conformation (276, 278). The exo-8, 9-epoxide metabolites of AFB1 are surprisingly prone to construct DNA adducts. This AFB1 metabolite is highly reactive, and its genotoxic effects have been well-documented (276, 278). Aflatoxin-N7-guanine metabolite also binds to the DNA, induces transversion mutations (pyrimidine ⇆ purine), affects the expression of tumor suppressor proteins and transcription factors, and ultimately dysregulates cell cycle events (276, 278). Acute exposure to AFs pollutants disrupts the fundamental function of genes involved in the glutathione pathway (282, 283). Studies reported that AFs could dysregulate the cellular levels of *PKC, PKA, p53, CDK, NF-*κ*B, Bcl2, CKI*, and cyclins (276). Disruption of mitochondrial function, ATP synthesis, and mitochondrial gene expression profile have been observed as side effects of AFs in the animal and human bodies (276).

The growing evidence suggests that AFs also influence the expression of xenobiotics metabolism genes, leading to upregulation of *CYP3A4* and pregnane X receptor (PXR) (275). In this regard, scientific outcomes have proven that the activation of PXR upregulates phase I and II metabolism genes and proteins such as *CYP2B6, CYP2C9, CYP3A4, CYP3A7*, UGT, GST, and SULT enzymes (275, 284). The dysregulation of *MDR1* and *OATP2* genes after activation of PXR has also been reported in several studies (275, 285). Studies reported that in primary liver cancer and cirrhosis, these genes are significantly overexpressed, leading to highlighting their critical role in the pathogenesis of cancer (286, 287). Considerable number of scientific investigations also demonstrated that exposure to AFB1 could disrupt the expression of *ERK, PKC-*β*, COX-2, caspases3/7/9, ASK1, SAPK, STAT3, E2FA, MYC, Bax/Bak, PUMA, CDKN1A, p21*, DNA/RNA polymerases, *PLK, MAPK*, and TRPs signaling pathways (288–291).

Additionally, continuous exposure to AFB1 promotes epigenetic modifications in liver cells (292). As detailed in the literature, AFB1 triggered various epigenetic alterations such as increased levels of aberrant DNA methylation, histone post-translational modifications, and up/down-regulation of non-coding RNAs (ncRNAs) and transcription factors (TFs) (293). AFB1-induced epigenetic drivers in the liver cells are associated with the development of hepatocellular carcinoma (14). Both AFB1-Lysine-protein and AFB1-DNA adducts inhibited the fundamental molecular process of infected cells by preventing transcription/translation of target genes. AFB1-based epigenetic alterations potentially increased the level of genomic mutations, inhibited the interaction TFs-gene-promoter complexes, modified the normal pattern of ncRNAs expression (e.g., miR34a/21/221 and lncRNA-H19), and altered signaling pathways (14, 293).

The evidence suggests that AFs strengthen the consequences of hepatocellular carcinoma risk factors such as DM, obesity, over-drinking alcohol, and viral infections (HBV, HCV) to influence the onset and progression of this catastrophic diseases (294). On the other hands, studies proven that AFB1/M1 end effects are not limited to liver cells so that it was shown that these AFs could also influence the metabolic profile of kidney (295). For example, in CD-1 mice co-treated with AFB1+AFM1 (0.5 mg/kg + 3.5 mg/kg) for 35 days, the results manifested that AFB1/M1 promoted the onset of oxidative stress in mice kidney, altered proline dehydrogenase and L-proline levels, sequentially induced upstream apoptosis, in turn, leading to kidney damage (295).

Evaluation of PPI networks of genes/proteins involved in AFs and xenobiotics metabolism unraveled that these genes (or proteins) constructed a network of interactions with various key proteins in cancer-linked signaling pathways. Up or down-regulation of these genes provides a ground for toxicity of AFs. In the human body, genes including *CYP1A2, CYP3A4, CYP2A13, GSTT1, GSTM1, EPHX1, AKR7A2*, and *AKR7A3* are driving AFs metabolism (296). The upregulation of these genes affects the expression of upstream/downstream genes, leading to a remarkable disturbance of cancer-associated signaling pathways (296) (Supplementary Figure S2). Studies have also shown that genetic polymorphisms in the structure of dominant genes involved in AFs metabolism might increase the risk of developing cancer (297).

Due to the presence of complex interactions between genes, proteins, and transcription factors in the human body, modification of gene expression levels after exposure to AFs can surprisingly affect the genotoxic, immunosuppressive, and mutagenic properties of these fungal metabolites (278). However, our literature searches showed that AFs interfere with DNA integrity, increase the secretion of pro-inflammatory factors, cytokines, and chemokines, inhibit DNA repair mechanisms, induce genomic instability, increase lipid peroxidation, induce DNA damage, cause tissue necrosis and organ failure, and dysregulate innate and adaptive immunity (290, 298–300). Reactive metabolites generated from AFs metabolism are highly toxic for body tissues, leading to obvious oxidative stress and the accumulation of ROS/RNS radicals (301).

Indeed, DNA-AFs structures can inhibit protein synthesis by disrupting the interaction of transcription factors and polymerase enzymes with DAN or increasing the occurrence of mutation in promoters and coding sequences (290). Considering the PPI networks, it is trustworthy to note that the dysregulation of the AFB1 metabolism network might be associated with disruption of gene expression networks in hepatocarcinoma, colorectal, pancreatic, melanoma, thyroid, bladder, and other types of cancers (Supplementary Figure S3). It seems that chronic exposure to AFs expedites a wide range of molecular irregularities in cells, ultimately leading to the pathogenesis of cancer, and more probably other nCDs.

## Cross-links between exposure to AFs and the onset of NCDs

According to WHO statistics (302), nCDs are accounted for more than 41 million deaths yearly, in turn, 77% all deaths occurred in low or middle-income countries (302). Environmental and genetic risk factors, such as exposure to biological toxins, pesticides, air pollutants, smoking, alcohol, unhealthy diet, cholesterol, obesity, physical inactivity, mental stress, work tension, elevated blood glucose, and blood pressure are common factors in triggering nCDs (303–306). Studies manifested that withdrawal of environmental risks factors and decreasing the exposure rate to health hazardous substances are effective strategies in preventing nCDs (306).

In the case of AFs, however, it is worthy to point out this fact that the elimination of these mycotoxins in food/feed is impossible, leading to the continuous existence of these risk factors in the environment. As evidenced in the previous sections, exposure to AFs is associated with development of liver cancer, though its role in the progression of MetSys and neurodegenerative diseases has not extensively been investigated. Therefore, this part of our review, by focusing on data obtained from high-tech omics-assisted outputs, describes how AFs may alter biological networks and/or gene expression profile being effective in the onset and development of nCDs.

Integrated transcriptomics and metabolomics analyses conducted on male Fischer rats given 0.25–1.5 mg/kg/b.w. AFB1 for 7 days reported that exposure to low-high concentrations of AFB1 is associated with dysregulation of tumor suppressor genes (at least 27 critical genes were up/down-regulated), antioxidant enzymes, cyclins, cyclin-dependent kinases, cytokine receptors, and inflammatory signaling pathways (307). According to this finding, acute exposure to AFB1 resulted in p53-induced oxidative damage, dysregulation of gluconeogenesis, and lipid metabolism, in turn, leading to hepatotoxicity of AFB1 (307).

In ducklings exposed to 0–40 μg/kg/b.w. AFB1 for 2 weeks, the RNAseq data disclosed that at least 749 transcripts responded to chronic exposure to AFB1 (308). Interestingly, these genes were critical components of phase I/II metabolism (*CYP1A5, CYP2H1, CYP2K1, CYP2F3*, etc), antioxidant enzymes (*GST1/3/K1*), fatty acid metabolism (*ACAA1, ACOX1, ACAT1, FADS1, FASN, HADH*, etc), apoptotic genes (*CASP3, CBR1, CCBL1, PPIF, KRT18*, etc), protein kinases (*PLK2*), oxidative responsive genes (*AKR1A1, AR, AO, FMO3, GPX4, NQO1, TXN, TDO2*, etc), cell cycles and cancer-associated genes (*PRELID1, PLK2, UGT1A8, MDM2*) (308). This study manifested that phase II detoxification enzymes such as *GST1, GSTK1, GST3* were up-regulated under chronic exposure to AFB1 (308), though previous studies found that these genes were down-regulated or not significantly influenced due to the difference between animal models selected for omics-based investigations (308, 309).

In Wistar male rats received 100–200 μg/kg intraperitoneal AFB1 for 4 weeks, the high-throughput gene expression analyses showed that exposure to AFB1 altered gene and lncRNAs expression (310). According to this finding, the identified differentially expressed lncRNAs were associated with upregulation of genes involved in cancer, apoptosis, DNA repair, and cell cycle arrest (310). Because several genes such as *Bcl2, MAPK8*, and *NFKB1* were up-regulated after exposure to AFB1, therefore, it has suggested that apoptotic-associated responses to AFB1 exposure played a critical role in hepatotoxicity of this mycotoxin (310). Similarly, in another omics-assisted study on chickens, the outcomes showed that exposure to AFB1 dysregulated genes involved in apoptosis and lipid metabolism in liver (311). This finding highlighted that *Bcl-6* gene was down-regulated whereas *PPARG* was up-regulated, in turn, might be leading to hepatic fat deposition and hepatocellular apoptosis (311).

The whole transcriptome of BFH12 (bovine fetal hepatocyte cell line) exposed to 0.9–3.6 μM AFB1 for 48 h displayed that AFB1 significantly dysregulated the expression of genes involved in inflammatory responses, apoptosis, oxidative stress, cancer and xenobiotics metabolism (312). Indeed, this investigation disclosed that exposure to low-high concentrations of AFB1 markedly influenced the activation of TLR2, p38β MAPK, AP-1 and NF-κB signaling pathway and pro-inflammatory cytokines (312). This outcome is important because a large quantity of studies published on toxicity of AFB1 for the animal and human bodies addressed oxidative stress and inflammatory responses as health consequences of this carcinogenic mycotoxin.

Bao et al. (313) reported that in Caco-2 cell lines exposed to 0.0005–4 μg/ml AFM1 for 48 h, totally 165 genes were down-regulated after exposure to AFM1. This finding demonstrated that exposure to AFM1 is associated with dysregulation of CDK1, AMPK, SOS1/Akt signaling pathways that are involved in cell cycle arrest (313). The metabolomics and transcriptomics analyses of mice liver and serum showed that co-exposure to AFM1 + OTA (3.5 mg/kg/b.w. for 35 days) significantly affected the phase I metabolism enzymes (ALT, AST, glutamyltransferase) by increasing their levels (314). Additionally, due to synergistic side effects of AFM1 + OTA, the accumulation of lipid droplets and liver steatosis were observed in co-treated groups (314). The metabolome profile of groups co-exposed to AFM1 + OTA disclosed that in liver and serum lysophosphatidylcholines levels were significantly increased (314). In another transcriptomics study conducted on wild and domesticated turkey exposed to 0.015–0.32 mg/kg AFB1, a significant dysregulation in phase I and II metabolism, inflammatory responses, and apoptotic genes was observed, in turn, provided another evidence for side effects of AFs on cellular and molecular targets (315).

As evidenced in these findings, chronic and acute exposure to AFs lead to remarkable alterations in cellular and molecular pathways. Based upon the given standpoints in the previous sections, it is important to note that continuous exposure to low-high doses of AFs certainly promotes the onset and progression of nCDs in societies with higher risks to encounter AFs in food/feed. These studies also addressed the role of inflammation, apoptosis and oxidative stress in progress of AFs-induced consequences, which in turn, can be considered as a scenario to characterize the susceptible signaling pathways and enzymes to develop future clinical management strategies.

**Figure 10:**
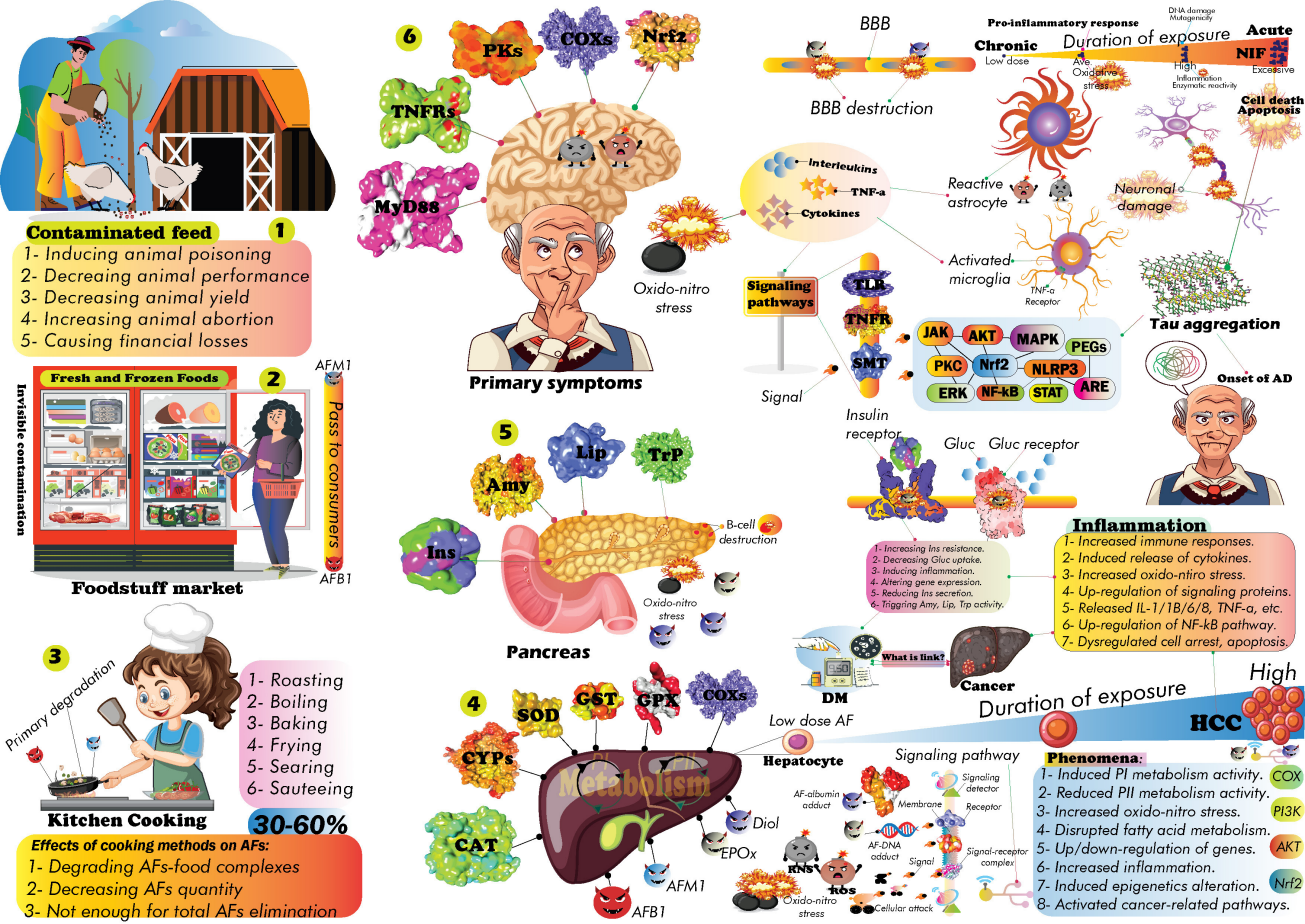
The cross-links between exposure to AFs and the progression of nCDs. Contaminated feed caused damages to animal farms and produced products will pass to the consumers (Steps 1–2). Food processing methods can effectively be used to eliminate nearly 30–60% of total AFs based on the cooking method (Step 3). The rest of AFs residues in food will later affect critical organs to trigger the onset of nCDs (Steps 4–6). Duration of AFs exposure plays a critical role in the pathogenesis of AFs side effects by affecting signaling pathways, triggering inflammation and oxido-nitro stress.

On the other hand, the growing body of evidence postulates that the public health impact of climate change is negative, might leading to expedite the onset of nCDs (316). Climate change alone is one of the most important drivers of AFs production under future conditions (72). Other climate change associated environmental risk factors such as improper storage of crops, infestation of secondary pests/pathogens, humidity and temperature can strengthen the production of AFs and other carcinogenic mycotoxins (53, 62, 72). Therefore, more contaminated foodstuffs will be entered local and international food chains, in turn, probably leading to increased risk of nCDs (305). The high-throughput outcomes of multi-omics-based data disclosed that AFs mainly have impact on the inflammatory responses and antioxidant defensive system. In HCC, however, dysregulation of inflammatory responses and antioxidant enzymes promoted the onset of hepatocarcinoma cancer (317–319), though these factors function in corporation with a complex network of protein-gene interactions. However, for other nCDs such as DM and AD, the mechanism underlying inflammatory responses, antioxidant enzymes, and cellular signaling cascades is markedly a sophisticated process and dysregulation of these pathways has a significant negative effect on the progression of diseases. Therefore, elimination of AFs risk factors in food/feed might, at least, provide a ground to decline the onset of nCDs. Figure 10 represents a cross-link between exposure to AFs and the onset of nCDs. An exciting point should be highlighted that is nCDs such as cancer, AD, and DM shared a commonality in the occurrence of oxidative/nitrosative stress and inflammatory responses. As shown in Figure 10, the onset of inflammation is the core component of these nCDs, in turn, leading to cellular apoptosis, dysregulation of enzymatic activity and ultimately the severity of nCDs pathogenesis.

## Phytometabolites interventions in AFs-induced health problems

According to FDA guidelines (320, 321), there has been no medicine to prevent the poisoning of AFs, though several protective interventional substances (e.g., NovaSil clay, chlorophyllin, oltipraz, sulforaphane, and tea PPs) have been addressed (322). Safe withdrawal of AFs, using modern agricultural practices, improving food processing methods, and preventing the consumption of contaminated foods have been reported as scenarios to reduce AFs quantity in food commodities (320–322). Recent findings suggested that the supportive and symptomatic care is a reliable health management strategy to reduce the poisonous effects of AFs (323). Correspondingly, using specific carbohydrate-rich and protein-restricted diets followed by administration of vitamins (e.g., B and K) can be helpful in the suitable prevention of AFs end effects (323). However, preventative methods should be cost-effective, and available for all individuals. By considering the link between climate change, onset of nCDs, and AFs health complications, it can be said that phytochemicals and plant-based products are contemporary solution in preventing the poisonous effects of AFs.

It is now well-documented that plant secondary metabolites are health promising compounds in the cornerstone of human diseases prevention (324). Medicinal herbaceous metabolites are ubiquitously found in fruits, vegetable, and even inedible plant materials (324). Efforts to reduce the complications of AFs demonstrated that plant-based products such as extracts, teas, essential oils, pure metabolites can decrease the toxicity of AFs by maintaining cellular normal function (324, 325). These herbaceous materials not only display health promoting properties in detoxification of AFs, but also prevent the formation of AFs in producing molds (326). Considering the link between exposure to chronic/acute doses of AFs and progression of nCDs, and also paying attention to this fact that there is no antidote to treat AFs poisonous effects (323), screening natural products pools to identify anti-AFs substances can alternatively help scientists in developing practical medicines against these biological carcinogens (20). Recently, the potential benefits of plant extracts and naturally occurring phytochemicals in mitigating mycotoxins consequences has been partially reviewed in the literature (324). Accordingly, herbaceous products could show rigorous antifungal activities to improve enzymatic function in degrading AFs (324) and enhancing their metabolism.

Despite the effectiveness of phytochemicals in reducing the severity of AFs side effects, there have been no comprehensive clinical studies to use these herbaceous metabolites for detoxifying of AFs. However, among natural products PPs are presently taken into consideration to detoxify AFs, and their health benefits expedited scientific investigations in this respect (191, 327–336). The popularity of PPs can be attributed to their spectacular antioxidant and biological properties (337, 338). According to our literature searches, hundreds of studies have been conducted on anti-AFs properties of PPs to show how these phytochemicals might ameliorate the toxicity of AFs in the animal body (339, 340). Previous studies substantially confirmed the health promoting impacts of PPs in alleviating MetSys (341), neurodegenerative (342) and chronic diseases (343).

## General overview of PPs chemistry

PPs are a diverse category of plant secondary metabolites, displayed health-promoting properties, and are marketed as dietary supplements (338, 344, 345). Originally, PPs are defensive metabolites in plants secreted in response to abiotic and biotic tensions (346). It is now well established that the regular consumption of PPs and/or PPs-rich foods are linked to a healthy lifestyle (347–349). To date, the chemical structure of more than 8,000 PPs has been characterized, and the emerging evidence suggests that global demands for PPs-rich foods (or supplements) have increased over the past decades (344, 347).

PPs could scavenge free toxic radicals and display potential antioxidant activity (350). Evidence-based data reported that PPs showed no toxicity effects on the human body (338, 350). These metabolites mainly function as potent anti-bacterial, antiviral, anti-diabetic, anti-cancer agents, and are modulators of molecular signaling pathways (351, 352). A plethora of studies has been conducted on PPs to modify their structure in developing reliable medicines for treatment of human diseases (353). After ingestion of PPs-rich foods, these metabolites are metabolized in liver, enter the blood circulation system and are transported to different parts of the human body (345).

The chemical backbone of PPs consists of at least one aromatic ring and several OH groups. In this regard, PPs can be classified into two main groups, including flavonoids and non-flavonoid metabolites (346). Simple PPs mainly shared C1–C6 and C3–C6 backbones. These PPs have a low molecular weight and are widely biosynthesized in flowering plants. Instead, flavonoids share a C6–C3–C6 backbone, and two benzene rings are existed in their structure (354). Flavonoids are among the most abundant and well-studied PPs, displayed potential antioxidant and health-promising effects. This class of PPs divided into several subgroups with distinct biological and chemical features (355).

**Figure 11:**
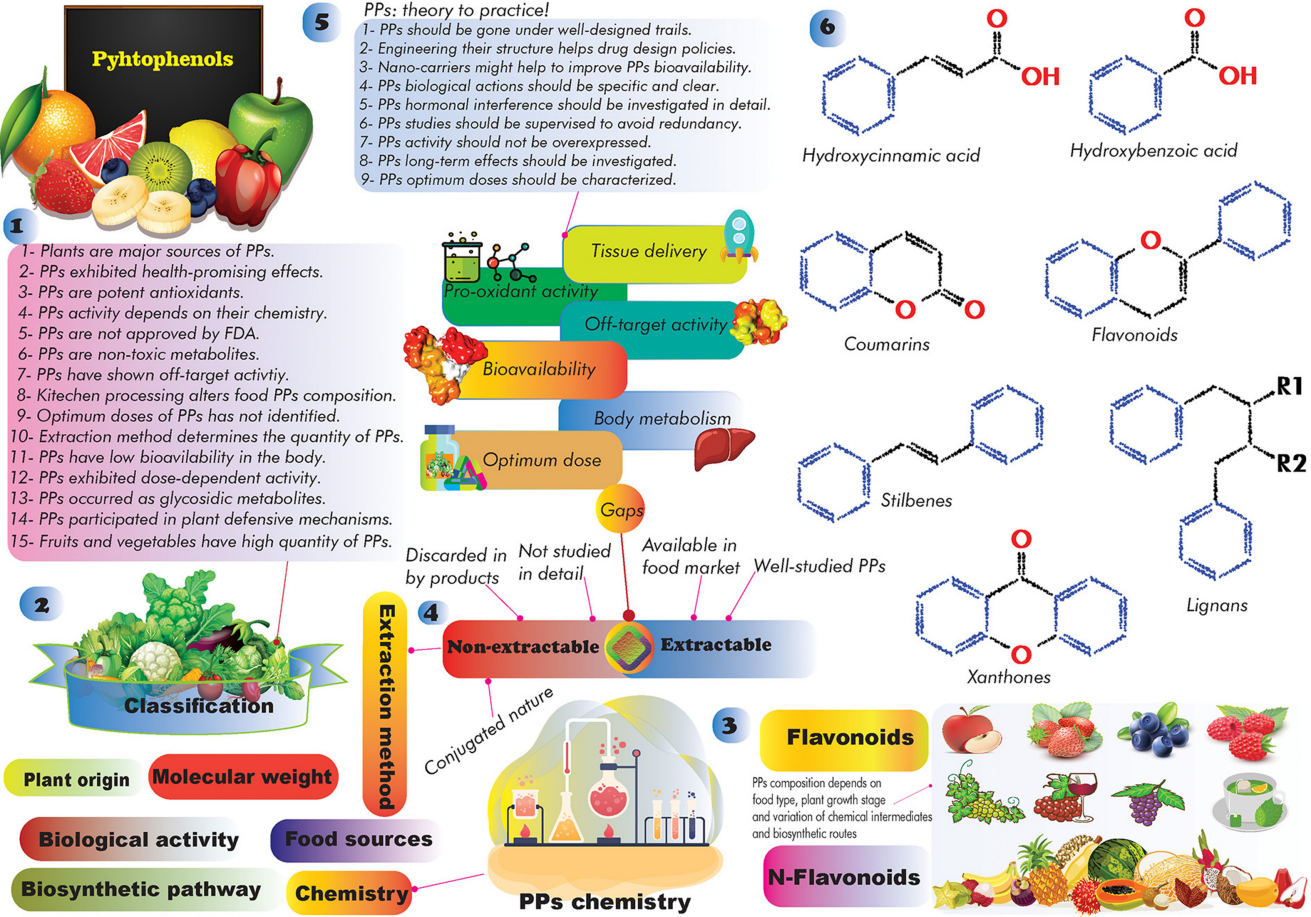
A quick glance at PPs. (1) general facts about PPs, (2–4) PPs classification systems and relevant gaps, (5) PPs ignored facts, (6) the core backbone of well-known PPs. R1 and R2 represent the chemical moieties prone to bind these positions.

Studies have shown that the biological activity of flavonoids is associated with the substitution of functional chemical moieties on their backbone (356). In nature, flavonoids and other PPs mainly occur in the form of O-glycosylated metabolites. These highly hydroxylated PPs are prone to other chemical modifications such as methylation and acetylation (347). The review of the literature showed that the majority of studies on PPs targeted O-glycosylated PPs, while the biological properties of C-glycosylated PPs have remained relatively unclear (357, 358). Stilbenes, lignans, curcuminoids, coumarins, and xanthones are other classes of non-flavonoid metabolites with potential biological activities (345, 347, 359). Figure 11 summarizes relevant information on PPs.

## Using modern biotechnology for ME of PBPs

The advancement of biotechnological methods supports researchers to integrate new genes, and other interested molecular components into target organisms for improving their qualitative and quantitative traits (360, 361). Genetically modified organisms have a particular gene expression profile in which genes might be silenced or over-expressed to obtain the desired traits (362–365). In this regard, ME of PBPs received much attention from academic and industrial sectors due to health benefits of PPs (366). Today, sufficient pieces of information about PBPs are available to introduce their biosynthetic genes into new hosts for overproduction of these highly valuable metabolites (360, 366).

The ME of PBPs can be performed in different ways, including overexpression of PPs biosynthetic structural genes and transcription factors, up and down-regulation of genes/enzymes involved in the biosynthesis of certain PPs, silencing of specific metabolic routes in phenylpropanoid pathway, down-regulation of PPs biosynthesis structural genes using interference miRNAs, and enhancing the production of intermediary substrates of phenylpropanoid enzymes (365–369).

In this regard, several lines of evidence suggest that the metabolic engineering of PBPs could enhance crop resistance against plant pathogens (369, 370). In an interesting study, ME of PBPs in soybean hairy roots influenced root resistance to fungal pathogens (370). Additionally, scientific outcomes also reported that the presence of gallic acid and hydrolysable tannins in the pellicle tissues of walnuts were accounting for prevention of AFs biosynthesis (371). Biofortification lignin biosynthesis in plants has been considered as a defensive strategy in the retardation of pathogen entrance and growth (372). For example, the overexpression of *OsWRKY89* gene in GM rice plants has shown to influence resistance to rice blast by modulating the biosynthesis of PPs and increasing the lignification process of GM lines (373).

The overexpression of *PAL* gene in GM tobacco plants could enhance their resistance against *Phytophthora parasitica* and *Cercospora nicotinae* fungal pathogens (372, 374). In this respect, studies manifested that transformation of other key regulators of phenylpropanoid pathway along with *PAL* gene might increase phenolic content (in particular rutin and chlorogenic acid) of GM plants compared to wild-type lines (375). These outcomes, as documented in the literature, indicated that production of PPs-rich GM plants not only improved plant resistance to fungal pathogens, but also enhanced their antioxidant properties (372). Correspondingly, engineering of flavonoids pathways in flax plants has shown to increase the accumulation of fatty acids in GM flax seeds and oil (376). This outcome has shown that the overproduction of flavonoids resulted in the prevention of lipid oxidation during seed development and maturation and increased its antioxidant properties for biomedical applications (376). Multi-level engineering of tomato plants by targeting specific transcription factors (e.g., *AtMYB12*) has been increased the fruit dry weight, carbon metabolisms, and improved the functionality of shikimic acid and phenylalanine pathways (377). This outcome indicated that the accumulation of phenylpropanoid bioactive metabolites not only improved the quality of engineered tomato plants, but also provided a ground for biofortification of healthy foods enriched in health promoting secondary metabolites (377).

The overexpression of maize *Lc* gene in transgenic apples spectacularly increased the production and accumulation of anthocyanins and falvan-3-ols resulted in a higher resistance to *Erwinia amylovora* bacterium and *Venturia inaequalis* fungus (378). In another line of research, the cloning of *Solanum sogarandinum* 7-O-glycomethyltransferase enzyme in transgenic flax plants significantly increased the accumulation and stability of flavonoid glycosides (anthocyanidins and flavonols), leading to a higher resistance to the fungus *Fusarium* and improved antioxidant and oil content of GM flax (379). Further information regarding scientific investigations on ME of plants PBPs can be obtained in the available literature reviews (366, 367). Indeed, Microbial production of PPs using GM bacterial strains have also resulted in significant over-production of these health promising metabolites (360, 380) which can be used to expand clinical and experimental studies on PPs.

The available evidence suggests that such biotechnological methods not only increased the quantity and availability of PPs for industrial, food and medicinal purposes (360, 381, 382), but also expedited the number of scientific investigations conducted on PPs to find the most potent metabolites for large-scale applications (361). The available literature suggests that there have been no comprehensive studies in the literature to engineer crops PBPs against APF. Therefore, developing highly PPs-rich GM plants among susceptible crops to AFs using modern biotechnological and breeding procedures might be considered as alternative mycotoxin management strategy to decrease the global spread of AFs. Although presently GM crops are not universally popular due to the rumors revolved around (383, 384), nevertheless, GMOs are part of hundreds of suggestions to eliminate AFs in food/feed. Additionally, the ME of PPs biosynthetic pathways might increase the long-term resistance of recombinant plants to invasive fungal pathogens and decrease the application of chemical fungicides, though this benefit will require further large-scale investigations to confirm its efficacy against crop fungal pathogen damages.

## PPs as modulators of critical signaling pathways

As anticancer agents, PPs could modulate signaling pathways involved in cancer by up and down-regulation of gene expression and suppression of the release of inflammatory factors (385, 386). Experimental assays also unraveled that PPs could inhibit cancerous tumor growth by triggering apoptosis and blocking signaling pathways involved in tumor cell angiogenesis (387). The antioxidant content of PPs also support these metabolites in decreasing ROS/RNS levels in cancerous cells (388). Indeed, PPs affect the cancerous cells gene expression profile, leading to suppressing cancerous cell development and metastasis (389, 390). The growing body of evidence suggests that PPs have impacts on DNA methylation and epigenetic modifications associated with progress of cancer (390). PPs also inhibited the activity of DNA methyltransferases, leading to significant changes in the methylation pattern of specific genes involved in various types of cancers (391). PPs could also modify the expression of microRNAs involved in the regulation of cancer metastasis pathways (392).

Proteins such as G protein-coupled receptors, PI3K, AKT, MMPS, EGFR, VEGF, ERK, STAT3, p53, FOX, JNK, caspases, JAK, PKC, FGF, Nrf2, ALK, ROS1, mTOR, and MAPK are pivotal derivers in the pathogenesis of human cancers (386, 392). In a dose-dependent fashion, PPs could modulate the activity of these proteins, leading to inhibition of primary phases of tumor development (386, 393). Blocking and/or modulating the secretion of cancer-induced pro-inflammatory mediators is another health-promising effect of PPs (394). Inhibition of cancerous cell proliferation (395), inducing apoptosis (387), and suppressing cell cycle events are promising strategies in the cornerstone of cancer therapies. PPs have been shown to regulate these critical processes in cancerous cells through a range of molecular mechanisms (396). Anticancer activity of PPs in clinical trials and animal studies has also substantially studied (397–400).

As evidenced in the literature, as anti-inflammatory phytochemicals certain PPs (e.g., flavonoids, stilbenes, curcuminoids) could ameliorate the consequences of neuroinflammation (248). These phytochemicals principally interacted with pivotal neuro-inflammatory signaling waterfalls, improved brain enzymatic activity, decreased nitrosative stress and RNS formation, improved brain antioxidant defense, regulated of pro-inflammatory-related gene expression, restored the activity of astrocytes and microglial cells, modulated brain transcription factors expression, alleviated COXs expression, enhanced expression of anti-inflammatory genes, and protected brain neuronal cells (248, 401, 402).

As anti-diabetic phytochemicals, PPs showed a broad-spectrum of biological activities to alleviate the complications of DM (403, 404). In this regard, several studies generally attributed the anti-diabetic potential of PPs to their capability to reduce blood glucose level, improve insulin sensitivity and secretion, alleviate oxidative/nitrosative stress, inhibit carbohydrate digestive enzymes, alleviate β-cells apoptosis, ameliorated lipogenesis, alleviate glucogenolysis and gluconeogenesis, up- and down-regulate of DM-associated genes, and modulate signaling pathways (NF-κB, ERK, PPAR, AMPK, cytokines, protein tyrosine phosphatases, glucose transporter receptors, hepatic enzymes, tyrosine kinases, insulin receptors) (403–406).

Biochemical and metabolic factors, including concentrations of PPs, duration of PPs administration, PPs metabolism and post-metabolism modifications, interaction with intestine metabolites/enzymes, interaction with gut microbiota, gastrointestinal uptake, and their bioavailability the body tissues affect the molecular effects of PPs. The following sections summarized the recent trends on the application of PPs in prevention of AFs consequences.

## PPs mechanism of actions for improving AFs complications

As documented in the literature, PPs exhibited anti-fungal activity against various types fungal pathogens (407). Respectively, Ahmed and colleagues comprehensively reviewed the inhibitory profile of PPs in preventing AFs production (407). This study, however, focused on PPs mechanism of actions to inhibit AFs formation, and highlighted the associated food safety issues (407). Indeed, another review by Fan et al. summarized recent updates on the application of phytochemicals in detoxifying AFB1-induced hepatotoxicity (21). This study also included PPs as possible candidates to ameliorate AFB1 side effects, however, the study mainly discussed anti-AFB1 mechanisms of different phytochemicals (21). There have not been similar comprehensive review studies on anti-AFs activity of PPs in the literature until the time we prepared this review. Therefore, to increase our current knowledge of anti-AFB1 properties of PPs, in this section we provided an in-depth insight into PPs/PPs-rich extracts mechanism of actions in alleviating health hazardous effects of AFs, in particular AFB1, by summarizing recent trends obtained from animal-based and *in vitro* studies.

Due to multiscale biological activity of PPs, scientific investigations promoted these naturally occurring metabolites in preventing AFs complications (324, 408). Studies suggested that PPs can directly or indirectly affect the metabolism of AFs, leading to significant reduction of AFs toxicity (409–414). These interesting results purported that PPs substantially interfered with the formation of AFs-HSA complex (191), reduced the construction of AFs-DNA adducts (415), regulated AFs-induced inflammation (416), and also improved detoxification of AFs in liver (417). Bearing this fabulous quote “*All things are poison, and nothing is without poison; the dosage alone makes it so a thing is not a poison*,” credited to Paracelsus (418) in mind, it can be said that only particular doses of PPs might suppress the onset of AFs-induced metabolic and chronic disorders. Therefore, understanding the biological properties of PPs after their metabolism and tissue intake in the body can supposedly help to identify the exact behavior of these metabolites in AFs therapy scenarios. In this regard, there are hundreds of studies confirmed that PPs could alleviate the end effects of AFs-induced complications, as detailed in the following paragraphs.

In rats fed with 72 μg/kg/b.w. AFB1 and 100 mg/kg/b.w. PPs-rich leaf extract of artichoke (*Cynara scolymus* L) (PLEA) for 42 days, the outcome manifested that the PLEA promoted partial neuroprotective properties. Accordingly, PLEA down-regulated total plasma lipids/LDL/VLDL, and simultaneously increased HDL levels (419). Although supplementation of PLEA alone had no effects on TNF-α, TIMP3 and IDO concentrations in the brain of rats, however, the results displayed that when rats supplemented with PLEA + AFB1 the total concentrations of these biomarkers relatively decreased but still was not meaningful compared to control group (419). Co-treatment of rats with PLEA +AFB1 was also ameliorated the oxidative stress in the brain of rats, and improved antioxidant enzymes. It seems that the joint administration of PLEA + AFB1 could alleviate oxidative stress in the brain and improve the histological effects of AFB1 on the brain (419).

Similarly, quercetin (30 mg/kg) showed significant neuroprotective effects in Balb/c mice co-administrated with AFB1 (0.75 mg/kg/b.w.) (328). Quercetin reduced TNF-α and IL-1β levels, increased GSH, CAT, and SOD levels, and prevented memory impairment in mice exposed to chronic levels of AFB1 (328). In rats co-supplemented with 80 μg/kg/b.w. AFB1 and 300 m/kg/b.w. PPs-rich ethanolic extract of *Chelidonium majus* (PEEC), the outcome showed that PEEC alleviated the neurochemical biomarkers (420). In AFB1-treated group, rats showed a significant increase in TNF-α, IL-1β and CD4, AChE, dopamine, and caspase 3 levels whereas co-treatment of PEEC (not PEEC alone) significantly alleviated the increased levels of studied neurochemical markers in rats' cortex and hippocampus areas, and improved the activity of antioxidant enzymes (GSH, SOD, CAT, GPx) (420).

As evidenced in the literature, gallic acid (GAc) abated the health consequences of exposure to AFs. In a study conducted by Owumi et al. (421), the evidence suggested that gallic acid exhibited potential anti-inflammatory and antioxidant activity in rats co-treated with 75 μg/kg AFB1 and 20–40 mg/kg GAc for 28 days. GAc exhibited its antioxidant activity by increasing GSH, SOD, CAT, GPx levels (421). This compound improved antioxidant balance in testes, hypothalamus, and epididymis of rats. The outcome also unraveled that GAc decreased lipid peroxidation, and oxidative/nitrosative stress (421). Correspondingly, GAc alleviated apoptosis mediators in rats by reducing IL-1β, TNF-α, nitric oxide levels, and suppressing myeloperoxidase (421). This observation proven that GAc could ameliorate AFB1-induced oxido-inflammatory responses in reproduction system (421). In Swiss male albino rats co-supplemented with 750–1,000 μg/kg/b.w./day AFB1 and 2 mg/0.2 ml olive oil/day, the outcomes showed that curcumin could ameliorate the AFB1-toxic effects in reproduction system of rats by improving caput and cauda epididymis weight and enzymatic activity (422). In a similar study, co-treatment of curcumin and AFB1 displayed that curcumin alleviated AFB1-induced toxic effects in rats' reproduction system by improving semen parameters such as sperm quality and quantity, mobility, viability and other sperm relevant features (423). In another study, the whole transcriptome analysis of BFH12 cell lines co-treated with curcumin and AFB1, the finding purported that curcumin abated the inflammatory responses and improved antioxidant enzymes in AFB1-treated cells (424).

In a dose-dependent manner, phenolic metabolites such as ellagic acid improved the activity of endogenous antioxidant enzymes, prevented DNA damage and exhibited antimutagenic properties in animal models exposed to AFB1 (324). Caffeic acid in the concentration of 40 mg/kg exhibited protective effects in reproduction system of male rats exposed to 50 μg/kg/b.w. AFB1 through modulation of antioxidant enzymes, apoptosis, and inflammatory factors (425). Apigeninidin-rich extracts of *Sorghum bicolor* L. Moench (ASBEs) including ASBE-05/06/07 modulated inflammation and apoptosis mechanisms in kidney and liver of rats exposed to 50 μg/kg doses of AFB1 (426). ASBE-06 with IC50 = 6.5 μg/ml suppressed lung cancer cell lines. Correspondingly, ASBEs were also modulated the expression of STAT3 and caspase 3 proteins and displayed protective role against oxidative and nitrosative stress (426). In an interesting study, total flavonoids extract of *Rhizoma Drynariae* in the concentration of 125 mg/kg inhibited AFB1-induced apoptosis and regulated the expression of *PI3K, AKT, Bax* and *Bcl2* in broilers chickens (427). In rats fed with 400 mg/kg/b.w. AFB1, oxidized tea phenolic compounds in the concentration of 100 μg/kg directly bound to AFB1, lowered its plasma level, and increased AFB1 fecal excretion (27).

Quercetin in the concentration of 50 and 100 mg/kg/b.w. displayed protective hepatocellular effects in liver of rats received 1.4 mg/kg/b.w. AFs-containing diet (428). This finding is in agreement with previous outcomes that examined the efficacy of different doses of quercetin (15–45 mg/kg/b.w.) in prevention hepatic damage of AFs in mice (327). Quercetin also showed protective role against DNA damage when HepG2 cells treated with 5 μg/ml quercetin and 1 μM AFB1 for 2 h, leading to a significant decrease in DNA damage from nearly 60–32% (21, 333). In an interesting investigation, chicks exposed to 5 mg/kg AFB1 displayed an alteration in the activity of AST, ALT, nitric oxide synthase, COX-2, caspase1/3/11, antioxidant enzymes, and pro-inflammatory factors such as TNF-α, IL-1β/6 (429). Morin, a flavonol derivative, in a dose-dependent manner (20–80 mg/kg) ameliorated inflammatory responses, restored AFB1-induced liver and kidney damages by modulation of gene expression and prevention of hepatocyte disruption in AFB1 fed chicks (429).

Kolaviron, a bioflavonoid extracted from Garcinia kola in mice administrated with 100 and 200 mg/kg of this compound and 2 mg/kg AFB1 for 4 weeks significantly reduced the AFB1-induced genotoxicity and oxidative stress (430). This bioflavonoid not only abated the total level of AFB1-DNA adducts, but also decreased the AFB1-induced GGT, AST, and ALT activity by 72, 62 and 56% (430). In adult rats treated with either 10 mg/kg/b.w. quercetin nanoparticles (QNPs) or quercetin and 80 μg/kg/b.w. AFB1, the outcomes have shown that QNPs showed significant anti-aflatoxigenic properties compared to pure quercetin (330). In this regard, QNPs (52.70 nm size) significantly reduced ROS formation, AST/ALT and alkaline phosphatase levels, improved cell viability, glutathione level, and mitochondrial function, and decreased lipid peroxidation (330). This outcomes suggested that Nano-formulation of PPs such as quercetin strengthened their hepatoprotective properties to alleviate the compilations of AFs (330). In rats administrated with subcutaneous 25 mg/kg/b.w. ternatin, a tetramethoxyflavone extracted form *Egletes viscosa*, and 1 mg/kg/b.w. for 72 h, the finding suggested that this bioflavonoid significantly reduced AFB1-induced AST/ALT levels, modulated MDA level, and displayed chemoprotective effects against liver injury (431). Ternatin markedly inhibited lipid peroxidation, bile-duct proliferation and hepatic necrosis as vitamin E did (431). In another study, the outcomes showed that ternatin decreased plasma liver GSH quantity, alleviated liver oxidative stress and glycemic state, but had no effects on liver regeneration (432).

As discussed formerly, AFs displayed a potent binding affinity to human HSA (191) as well as BSA (193). PPs such as resveratrol could compete with AFs to bind to critical active sites of HSA by which showed an influential effect to decrease the bioavailability of AFB1 and displace the stability of AFB1-HSA complex (177). Studies demonstrated that flavonoids generally displayed a moderate binding potency to HSA, however, flavones and flavonols disclosed a higher tendency to interact with HSA (433). Chemical modification (e.g., sulfation and glycosylation) of flavonoids backbone might also be effective in lowering or increasing their binding affinity to HSA (433). In a dose-time dependent manner, green tea PPs (GTPs) modulated the AFB1 metabolism (by inhibiting of phase I and inducing of phase II metabolism), and decreased the formation of AFB1-HSA adducts (408). Additionally, GTPs in the concentration of 500 and 1,000 mg significantly increased the excretion of AFB1–mercapturic acid, the metabolized by-product of AFB1-8,9-epoxide, into urine which indicated a significant modification in the activity of GSTs (408). The outcome of this clinical trial was also displayed that there was no significant change in urine AFM1 levels, however, this finding strongly supports the protective roles of GTPs in regulating AFB1 metabolism and detoxification (408).

Notably, AFB1 induced hepatocellular pyroptosis (434), and caused critical impairment of liver KCs (435). In mice treated with 1 mg/kg/b.w. AFB1 for 4 weeks, the outcomes showed that AFB1 activated NLRP3 inflammasome and inflammatory infiltration, up-regulated COX-2, enhanced the secretion of IL-1β, and activated KCs, leading to inflammatory-induced liver injury (434). The flavonoid silibinin displayed hepatoprotective activity via selective modulation of certain pathways in activated KCs isolated form rat liver (436). Accordingly, silibinin inhibited nitrosative and oxidative stress in a dose-dependent fashion (IC50 = 80 μM/L). While it has not inhibited prostaglandin E2, silibinin was effective in inhibiting of leukotriene B4 (IC50 = 15 μM/L) and 5-lipooxygenase pathway (436). Curcumin was also found to be functional in preventing hepatic pyroptosis and oxidative stress (416). In this respect, in mice given oral curcumin (100–200 mg/kg) and AFB1 (0.75 mg/kg) for 30 days, the outcome showed that this metabolite mitigated AFB1-induced inflammatory liver injury by inhibiting of NLRP3 inflammasome activation, enhancing phase II AFs detoxifying metabolism, up-regulating of Nrf2 signaling pathway, and preventing the release of pro-inflammatory IL-1β/18 (416).

An increasing volume of evidence suggests that flavonoid subclasses mainly attenuated toxin-induced liver injury by regulating MAPK/NF-κB, CYPs, TLRs, c-JNK/ERK, cytokines/chemokines, Nrf2/CYP2E1, Bcl2/AKT/caspases signaling cascades, preventing oxidative stress, and enhancing antioxidant enzymes (437). AFs has shown to induce the expression of *CYPs* genes (438). Studies also proven that PPs prone to interaction with CYPs isoforms, which in turn can decrease the biotransformation of AFs after ingestion (439). Generally, mechanism of AFs-induced liver injury is a sophisticated process. The accumulating body of evidence suggests that the liver toxicity of AFs is mainly associated with oxidative/nitrosative stress, cellular apoptosis, mitochondrial dysfunction, lipid/protein peroxidation, construction of AFs-DNA adducts, DNA damage, induction of genomic mutation, inhibition of tumor suppressor proteins, up-regulation of gene expression, induction of inflammatory signaling pathways (21, 440).

In this regard, robinetin and other polyhxdroxy flavonols inhibited microsome-assisted formation of AFB1-DNA adducts (334). Mutually, in Wistar male albino rats exposed to 2 mg/kg/b.w. AFB1 for 6 weeks, administration of 25 mg/kg/b.w. silymarin (or silibinin) could decrease lipid peroxidation and improve the activity of antioxidant enzymes of liver (up to 44–100%) and kidney (up to 82–100%) (441). It could also protect liver from the altered levels of DNA, RNA, glycogen and cholesterol by 70–100%, and hepatic GSH up to 25–37%, respectively (441). Grape seed proanthocyanidins (GSPAs) also showed protective role against AFB1-induced DNA damage. In male Swiss albino rats received 0.5–1 mg/kg AFB1 for 2 days and 100–200 mg/kg/day GSPAs, the outcome suggested GSPAs modulated the expression of *Ogg1, Parp1*, and *p53* genes involved in DNA repair (442).

PPs-rich cocoa extract has also been tested to investigate its anti-AFs properties (443). The outcomes suggested that it was not effective against AFB1 but it significantly reduced the ROS formation and increased cell viability in cells treated with AFB1 alone or mixture of AFB1 + OTA (443). On the contrary, flavonoids-rich fractions prepared from *Rhus verniciflua* Stokes (FRVs) displayed both *in vitro* and *in vivo* chemoprotective against AFB1-induced liver injury. FRVs remarkably decreased ROS formation and MDA level and improved cell viability in HepG2 cells (444). Correspondingly, oral administration of FRVs suppressed AFB1-increased serum level of ALT, lactate dehydrogenase and alkaline phosphatase. FRVs improved glutathione balance and superoxide dismutase activity in AFB1-adminstarted mice liver (444). Indeed, FRVs increased IgA and IgG titers in mice serum. Form this outcome, it can be concluded that FRVs increased the formation of AFB1-GSH complex and restored antioxidant defense (444). In male Wistar rats treated with 100 and 250 mg/kg ginger PPs-rich extract (GPE) and 200 μg/kg AFB1 on the basis of daily, the results showed that GPE could significantly reduce liver damage, AFB1-induced toxicity, and showed remarkable hepatoprotective properties (445). Additionally, it was observed that GPE could improve the activity of endogenous antioxidant enzymes, up-regulate Nrf2/HO-1 pathway, and reduce lipid peroxidation (445). In piglets fed with 8% PPs-rich grape seed extract (PGSE) and 320 μg/kg AFB1 for 30 days, the outcome showed that lower concentration of PGSE has a low to moderate impact on oxidative stress and inflammation in piglet spleen, suggesting that greater concentration of PGSE is required for better alleviation of AFB1 toxicity (446). The flavone aglycone diosmetin displayed anti-OTA activity in MDCK kidney cells by regulation of cellular ATP levels (447).

By-products of palm oil industry such as PPs-rich palm kernel cake (PPKC) also alleviated AFB1-induced cell damage and showed hepatoprotective effects in chicken hepatocytes (448). It is believed that the molecular mechanisms underlying anti-AFB1 properties of PPKC are associated with prevention of lipid peroxidation, modulation of pro-inflammatory and apoptosis genes, and improving the activity of antioxidant enzymes (448). A study has displayed that in rats administrated with 250 mg/kg Korean red ginseng (*Panax ginseng*) extract (KRGE) and 150 μg/kg AFB1, KRGE could alleviate the adverse effects of AFB1-induced inflammation and hepatotoxic effects (449). Accordingly, KRGE improved serum biomarkers and antioxidant enzymes and prevented apoptosis in hepatocytes and liver inflammation (449). The recent studies have shown that *P. ginseng* comprised various types of PPs in which ferulic acid, rutin, chlorogenic acid, gentisic acid, *p-*/*m-*coumaric acid, catechin, and kaempferol were the foremost domineering phenolic metabolites in different tissues of this plant (450, 451).

Grapefruit juice extract in the concentration of 100 mg/kg has displayed protective effects against AFB1-induced liver DNA damage in F344 rats treated with 5 mg/kg AFB1 by gavage (452). Correspondingly, the administrated extract remarkably reduced hepatic CYP3A content but had no effects on CYP1A and CYP2C quantities (452). Studies confirmed that flavonoids (in particular naringin a flavanone-7-O-glycoside), vitamin C and other organic acids are major metabolic components of this extract (453), accounting for its antioxidant and biological properties (454). In rats treated with different concentration of olive cake PPs-rich extracts (OCPEs) (0.2–0.5 ml), its nanoparticle-based formulation and 22 μg/kg AFB1 for 4 weeks, OCPEs and its nano-formulation improved the neurotoxicity of AFB1 in rats brain (455). In male Wistar rats given 20 g/kg basal diet bee pollen (BP) and 3 mg/kg basal diet AFs for 30 days, the BP could ameliorate the toxicity of AFs by increasing the proliferation of lymphocytes (456). Although this observed benefit of BP was attributed to minerals, vitamins, and amino acids, however, PPs are also accounting for 1.6% total BP metabolites (456), which in turn might affect the anti-AFs properties of BP. The evidence also suggests that honeybee propolis, a resinous mixture of phytochemicals such as flavonoids and non-flavonoid metabolites, vitamins B/C/E, amino acids and other aromatic metabolites (457) could alleviate the toxicity of AFB1 by improving the activity of cytochrome P450 in honeybees (458). In male rats received 50 mg/kg/b.w. Iraqi propolis and 0.025 mg/kg/b.w. AFB1, the propolis has found to be effective in the restoring AFB1-induced gastrointestinal damages (459).

In mice orally administrated with different concentration of AFB1 and 2% aqueous black tea extract (ABTE) (instead of drinking water) for 30 days, the outcomes showed that ABTE ameliorated the AFB1-induced lipid peroxidation in mice liver by increasing the activity of enzymatic and non-enzymatic antioxidant contents (460). The observed benefit is accounted for the fact that ABTE is a PPs-rich fraction which exhibited significant antioxidant activities (460). Similarly, in mice co-administrated with low/high doses of ABTE and AFB1, supplementation of AFB1 resulted in significant reduction of DNA, RNA, glycogen and protein contents, and increased phospholipase activity and cholesterol content (461). In this regard, the ABTE co-administration displayed a protective role in mice liver against AFB1-induced biochemical changes (461).

The co-supplementation of 2% ABTE, 200 μg/kg/b.w. curcumin in rats given 750 μg/kg/b.w. for 90 days, the outcomes confirmed that the co-administration of ABTE-curcumin displayed synergistic effects in alleviating AFB1-induced liver damages in rats (462). Correspondingly, ABTE-curcumin could improve liver architecture, activity of antioxidant enzymes, lipid profile (in particular lowering cholesterol content) and liver biomarkers (462). In rabbits treated with 5 g/kg/b.w. coumarin and 0.25 mg/kg/b.w. AFB1, coumarin improved body weight and carcass gains, and reduced the toxicity of AFB1-induced complications (463). As detailed, PPs could exhibit their anti-AFs activity in a concentration-dependent manner, thus optimization of PPs concentration for treatment of AFs therapies is the most pivotal step in experimental assays regarding this field. These outcomes together confirmed that these plant metabolites are promising substances to reduce the health consequences of AFs. Table 1 provides detailed information on the mechanism of action of anti-AFs properties of studied PPs.

**Table 1:** The possible anti-AFs mechanism of action of PPs.

**Class**	**Compound**	**AFs**	**Mechanism of action**	**References**
Flavonols	Quercetin	AFB1	• Improving antioxidant defense	(191, 327–336)
			• Inhibiting lipid peroxidation	
			• Improving the enzymatic activity of SOD, GSH, and CAT.	
			• Protecting brain cells from AFs-related oxidative damage and decreasing hepatocellular damage	
			• Decreasing the expression of *Bax* and *caspase 3* genes	
			• Regulation of cellular apoptosis.	
			• Inhibiting cytochrome P450 reductase	
			• Protecting liver cells from oxidative stress damage caused by AFB1	
			• Decreasing the binding affinity of AFB1 to HSA	
			• Activating the Nrf2-ARE signaling pathway	
			• Increasing hepatoprotective effect after combination with nanoparticles	
			• Decreasing DNA damage	
			• Inhibiting AFB1 binding to cellular DNA	
			• Preventing the biosynthesis of AFB1 by-product derivatives	
			• Decreasing the elevated activity of PKC	
			• Inhibiting the formation of AFB1-DNA adducts	
		AFM1	• Inhibiting the bio-transformation of AFB1	(464)
			• Decreasing the synthesis of AFM1 by regulation of related signaling pathways.	
			• Regulating the activity of GSH/GST enzymatic function	
			• Inhibiting the pro-oxidant activity of AFB1	
	Rutin	AFB1	• Preventing DNA damage by regulation of DNA-associated enzymes	(465, 466)
			• Displaying antimutagenic activity	
	Azaleatin	AFs	Not reported	–
	Fisetin	AFB1	• Modulating antioxidant enzymes	(332, 335, 410, 413, 467–469)
			• Modulating the expression of inflammatory factors	
			• Decreasing aflatoxin-related oxidative stress	
			• Regulating the expression of TNFα and IL1α proinflammatory cytokines	
			• Modulating the enzymatic activity of GST	
			• Inhibiting hepatocarcinogenesis by regulating inflammatory-based signaling pathways	
			• Inhibiting cytochrome c P450 reductase	
			• Preventing metabolic activation of AFB1 by-products	
			• Inhibiting the formation of AFB1-DNA complexes	
			• Modulating the enzymatic activity of protein kinases such as GSK3β, PDK, PKB, PI3K	
			• Suppressing Akt protein signaling pathway	
			• Displaying dose-dependent antimutagenic effects against mutagenicity of AFB1	
	Galangin	AFB1	• Inhibiting cytochrome c P450 reductase	(332, 470, 471)
			• Preventing AFB1 conversion to toxic metabolites	
			• Protecting DNA from damage	
			• Preventing metabolic transformation of AFs	
			• Improving the activity of detoxifying enzymes	
			• Reducing the hepatotoxicity of AFs	
	Gossypetin	AFs	Not reported	–
	Kaempferol	AFB1	• Inhibiting the chemical transformation of AFB1	(409–414)
			• Displaying antimutagenic effects against toxic derivatives of AFB1	
			• Preventing lipid peroxidation	
			• Attenuating liver cell apoptosis	
			• Scavenging free toxic radicals in metabolic pathways of AFs metabolism	
			• Improving body antioxidative system	
			• Inhibiting AFB1 bioactivation in liver	
			• Regulating cytochrome P450 activity	
			• Suppressing the formation of AFB1-DNA adducts	
	Myricetin	AFB1	• Regulating cytochrome P450 activity	(332)
	Morin	AFB1	• Improving kidney injuries	(332, 429, 472)
			• Regulating ALT and AST activity	
			• Improving hepatocyte disruption	
			• Attenuating inflammatory signaling pathways	
			• Improving renal cells necrosis	
			• Decreasing the level of MDA	
			• Regulating the activity of SOD, GSH, and CAT enzymes	
			• Decreasing the expression of TNFα, IL-6, IL-1β, iNOS, COX-2, caspase-1/3/11	
			• Inhibiting AFB1-induced heterophil extracellular traps release	
			• Regulating oxidative and inflammatory responses	
			• Improving complications of aflatoxicosis	
			• Preventing biotransformation of AFB1 metabolic derivatives	
			• Improving detoxification of AFs	
	Rhamnetin		Preventing biotransformation of AFB1 metabolic derivatives	(471)
	Natsudaidain	AFs	Not reported	–
	Kaempferide	AFs	Not reported	–
	Isorhamentin	AFB1	• Protecting cells against oxidative stress	(327, 473)
			• Inhibiting AFB1 genotoxicity effects	
			• Suppressing lipid peroxidation	
				
	Rhamnazin	AFs	Not reported	–
	Astragalin	AFs	Not reported	–
	Robinin	AFs	Not reported	–
	Spiraeoside	AFs	Not reported	–
Flavanones	Hesperetin	AFB1	• Displaying antimutagenic effects against AFB1 metabolic derivatives	(410)
			• Regulating cytochrome c P450 activity	
	Hesperidin	AFB1	• Inhibiting neural crest cells from apoptosis induced by AFB1	(474)
	Naringenin	AFB1	• Inhibiting bioactivation of AFB1	(413, 475)
			• Regulating the activity of cytochrome P450 isoforms	
	Naringin	AFB1	• Inhibiting the induction of liver carcinoma	(476)
	Poncirin	AFB1	• Attenuating cellular apoptosis	(411)
			• Displaying hepatoprotective effects	
			• Inhibiting lipid peroxidation	
			• Improving the activity of antioxidant enzymes	
			• Protecting effects against oxidative stress	
	Pinostrobin	AFs	Not reported	–
	Sterubin	AFs	Not reported	–
	Sakuranetin	AFs	Not reported	–
Isoflavones	Genistein	AFB1	• Mode of action similar to poncirin + antimutagenic effects	(411, 477)
	Glycitein	AFs	Not reported	–
	Daidzein	AFs	Not reported	–
Flavones	Apigenin	AFB1	• Displaying antimutagenic effects by regulating SOS enzyme activity	(411, 478–480)
			• Inhibiting metabolic activation of AFs	
			• Suppressing oxidative stress and apoptosis	
			• Showing hepatoprotective effects in combination with other PPs	
			• Preventing ROS formation and DNA damage	
	Luteolin	AFB1	• Displaying antimutagenic effects in combination with other PPs	(478, 480–482)
			• Attenuating oxidative stress and apoptosis	
			• Showing protective properties for liver cells	
			• Activating the Nrf2 signaling pathway	
			• Scavenging free toxic radicals	
			• Decreasing the expression of *Bax, caspase-3/9, Cytc* genes	
			• Increasing the expression of the *Bcl-2* gene	
			• Up-regulating HO-1, NQO1, GCLC, SOD1	
			• Improving liver injuries	
			• Preventing ROS formation and DNA damage	
	Tangeretin	AFB1	• Displaying differential inhibitory effects on cytochrome c P450 activity	(332, 483, 484)
			• Minor regulating of mixed-function oxidase system	
			• Inhibiting unscheduled DNA synthesis	
			• Showing synergetic inhibitory effects in combination with other PPs.	
	6-Hydroxyflavone	AFs	Not reported	–
	Jaceosidin	AFB1	• Displaying antimutagenic effects	(479)
			• Inhibiting metabolic activation of AFB1 metabolites	
	Eupatilin	AFB1	• Displaying a similar mode of action like Jaceosidin	(479)
	Chrysoeriol		• Displaying similar modes of action like Jaceosidin and eupatilin	(479)
Flavan-3-ols	Catechin	AFB1	• Displaying antimutagenic effects against carcinogens in a dose-dependent manner	(485–489)
			• Inhibiting CYP enzymatic activity	
			• Regulation of NADPH-CYP reductase activity.	
			• Forming chemical complexes with AFB1	
			• Preventing the formation of AFB1-DNA complexes	
			• Improving liver injuries and oxidative stress complications	
	Epicatechin	AFB1	• Showing a similar mode of action to catechin.	(27, 443, 485, 488, 490)
			• Regulating of CYP enzymatic activity.	
			• Displaying indirect protective effects against AFs-related oxidative stress.	
			• Preventing mycotoxin-based DNA fragmentations.	
			• Scavenging free toxic radicals	
			• Protecting kidney cells from cell death	
			• Displaying hepatoprotective effects	
			• Inhibiting gastrointestinal absorption of AFB1	
			• Improving liver injuries caused by AFB1	
	Epigallocatechin	AFB1	• Displaying a similar mode of action like tea PPs	(27, 485, 488)
			• Improving oxidative stress and liver injuries	
	Epicatechin gallate	AFB1	• Displaying a similar mode of action like tea PPs	(21, 27, 485, 488)
			• Alleviating oxidative stress and inflammation	
	Epigallocatechin	AFB1	• Attenuating oxidative stress	(21, 27, 488, 491)
	gallate		• Displaying antimutagenic effects in combination with other PPs	
			• Inhibiting the biosynthesis of AFB1	
	Epiafzelechin	AFs	Not reported	–
	Fisetinidol	AFs	Not reported	–
	Guibourtinidol	AFs	Not reported	–
	Mesquitol	AFs	Not reported	–
	Robinetinidol	AFs	Not reported	–
Anthocyanins	Cyanidin	AFB1	• Inhibiting the biotransformation of AFB1 in a dose-dependent manner	(194, 471, 492–494)
			• Showing no inhibitory effects against the formation of AFB1-8,9-epoxide	
			• Improving oxidative stress induced by AFs	
			• Inhibiting the DNA and protein synthesis by AFs	
			• Reducing DNA fragmentation induced by AFs	
			• Inhibiting caspase-3 activation	
			• Scavenging ROS produced by AFs metabolism	
			• Showing hepatoprotective effects	
			• Interfering with the interaction of AFB1 and HAS	
			• Increasing cell viability HepG2 cells treated with AFB1	
			• Improving the activity of antioxidant enzymes GST, GPx and GR	
			• Inhibiting DNA damage and oxidative stress induced by AFB1	
			• Showing antimutagenic potency against AFB1-induced liver damage in a dose-dependent manner	
			• Improving antioxidant balance of cells in combination with glycosidic derivatives	
	Delphinidin	AFB1	• Inhibiting the biosynthesis of AFB1 in *A. flavous*	(495–497)
			• Showing indirect antimutagenic effects against mutagen metabolizing enzymes	
			• Showing synergistic mode of action in combination with other anthocyanins	
			• Suppressing the genotoxicity of AFs	
			• Showing protective effects against colon carcinoma induced by AFs	
			• Modulating phase II metabolism enzymes	
	Europinidin	AFs	Not reported	**–**
	Pelargonidin	AFB1	• Inhibiting biosynthesis of AFB1 in *A. flavous*	(495, 497, 498)
			• Displaying hepatoprotective effects	
			• Activating phase II metabolism enzymes	
			• Modulating Keap1/Nrf2 signaling pathway	
	Malvidin	AFB1	• Inhibiting biosynthesis of AFB1 in *A. flavous*	(495, 498, 499)
			• Displaying weak inhibitory effects on mycotoxins complications	
	Peonidin	AFs	• Inhibiting the biosynthesis of AFB1	(495, 499–501)
			• Strengthening plant defense system against AFB1	
			• Displaying weak inhibitory effects on mycotoxins complications	
			• Partial inhibition of IL-8 secretion	
			• Displaying anticancer activity but not related to induction of cancer by AFs/mycotoxins	
	Rosinidin	AFs	Not reported	**–**
Miscellaneous	Curcumin	AFB1	• Improving oxidative liver damage	(417, 471, 502)
			• Inhibiting lipid peroxidation	
			• Regulating serum marker enzymes	
			• Modulating the expression of inflammatory factors	
			• Displaying hepatoprotective effects	
			• Decreasing the complications of AFs-related inflammation	
			• Regulating Nrf2/HO-1 signaling pathway	
			• Influencing the autophagy of hepatocytes	
			• Regulating the activity of GSH, SOD, CAT and GSH-Px enzymes	
			• Inhibiting the biotransformation of AFB1	
			• Inhibiting the formation of AFB1-8,9-epoxide	
	Coumarins	AFB1	• Inhibiting the biotransformation of AFB1 in a dose-dependent manner	(471)
	Resveratrol	AFB1/2	• Increasing N6-methyladenosine mRNA methylation	(177, 464, 503–511)
			• Displaying hepatoprotective effects by scavenging ROS molecules produced by AFB1 metabolism	
			• Inhibiting AFB1-induced apoptosis	
			• Regulating Nrf2/Keap1 signaling pathway	
			• Inhibiting lipid peroxidation and regulating CYP genes (e.g., *CYP1A1, CYP1A2*) expression	
			• Regulating ALT, AST, MDA, and GSH levels	
			• Displaying hepatoprotective effects against liver injuries induced by AFs	
			• Displaying antigenotoxic effects by preventing DNA damage and degradation	
			• Competing with AFB1 to interact with DNA scaffold	
			• Preventing chromone aberration	
			• Decreasing the oxidative stress induced by AFB2	
			• Decreasing blood urea nitrogen	
			• Improving phase II metabolisms enzymatic activity and modulating antioxidant enzymes, SIRT1 and NF-κB/NLRP3 signaling pathways	
			• Inhibiting biosynthesis of AFB1 in *A. flavous*	
			• Competing with AFB1 to bind to HSA subdomains	
			• Inhibiting reproductive toxicity of mycotoxins	
	Olive PPs	AFs	• Inhibiting biosynthesis of AFB1 in *A. flavous*	(512)

As detailed in Table 1, PPs ameliorate AFs toxicity in different ways. Accordingly, the anti-AFs activity of PPs mainly contributed to preventing oxidative stress and inflammatory responses, inhibiting mutations in DNA, regulating signaling cascades, modulating phase I and II metabolism enzymes, improving cellular antioxidant balance, and interfering interaction of AFs and HSA. These data showed that flavonoids, in particular oxidized tea PPs, were the most studied PPs in the prevention of AFs toxicity. Indeed, the anti-AFs activities of resveratrol and curcumin were also highly investigated. Interestingly, different classes of PPs exhibited a wide range of heterogenous biological properties against toxicity of AFs. Our review clearly disclosed that PPs targeted the core pathways (inflammation-based responses) in the pathogenesis of AFs. This interaction is important because not only in the onset of cancer, but also inflammation (in particular NIF) play a critical role in in the progression of neurodegenerative disorders and MetSys (248, 250, 256). Therefore, as modulator secondary metabolites, PPs have the potential to maintain the normal status of cell by regulating the onset of inflammation-assisted signaling pathways and preventing the development of nCDs (338).

## PPs-rich extracts for inhibiting AFs production

PPs also prevent the formation of AFs in target fungi (*A. flavus* and *A. parasiticus* strains) by modulating fungal transcription factors activity (513, 514). For example, water-soluble and methanol extract of peanut tannins were also inhibited *A. parasiticus* growth and impaired the formation of AFs in a dose-dependent manner (515). Studies have also shown that the type of PPs extraction methods might affect antifungal activity of these metabolites. In this regard, solid-phase extraction of PPs-rich citrus peel extract displayed up to 40% antifungal properties against *A. flavus* compared to crude extracts (516). Correspondingly, 300–400 mg/ml mandarin PPs-rich extract is enough to completely inhibit *A. flavus* growth depending on extraction method and applied solvents (516).

PPs-rich methanolic extract of *Zanthoxylum bungeanum* (a traditional Chinese food additive) with the IC50 2–4 μg/ml significantly repressed the AFB1 biosynthetic pathway (517). The omics-based analysis of this extract unraveled that it could show the anti-aflatoxigenic properties by down-regulating of the global regulators of AFB1 biosynthesis such as velvet complex proteins, *Medusa* and *brlA* genes, and GPCR/oxylipin-based signaling cascade (517). Intriguingly, PPs-rich olive processing wastes (POPWs) also showed differential anti-aflatoxigenic properties in a dose-dependent manner to inhibit the growth of APF (518). Of all POPWs, olive pomace extract displayed a higher anti-aflatoxigenic properties in comparison to olive leave and pomace olive oil extracts, respectively (518). 5′-hydroxy-auraptene, a coumarin derivative isolated from *Lotus lalambensis*, in the concentration of 40 μg/ml prevented the AFB1 production and exhibited potential antifungal activity against *A. flavus* by down-regulation of genes involved in different phases of AFB1 biosynthetic pathway and inhibition of conidial germination of this fungus by 60% (519). The outcomes also suggested that 5′-hydroxy-auraptene disrupted the structure of mycelia sugar units and up-regulated stress mediated transcription factors (*atfA* and *atfB*) up to 2 and 2.5 folds (519).

Buckwheat hull PPs-rich extracts (BHPEs) displayed a dose-dependent inhibitory profile against *A. flavus* growth and AFB1 biosynthesis (520). According to this outcome the higher concentration of BHPE was accounting for longer inhibition of AFB1 formation (520). In another study, the pau ferro (*Libidibia ferrea*), a Brazilian medicinal plant, ethanolic fruit extract has found to be effective in the inhibition of *A. parasiticus* growth (521). PPs-rich *Cistus incanus* L. methanolic extract in the concentration of 0.2 g/ml significantly decreased the formation of AFB1 production from 72.5 to 90.1% and inhibited *A. parasiticus* growth (522). The essential oil of *C. ladanifer*, another species of *Cistus* genus, has also found to be effective in the prevention of *A. flavus* growth by suppressing AFB1 production, and inhibiting the fungus ergosterol biosynthetic pathway with MIC value of 0.6 μl/ml, respectively (523).

An increasing trend of evidence purported that glycosidic and aglycone derivatives of flavonoids and non-flavonoids in a dose-dependent manner suppressed the production of AFB1 and other mycotoxins by targeting the critical routes in biosynthetic pathways of toxicogenic fungi (407). For instance, compound of interest, quercetin also disrupted AFs-producing fungal proliferation in addition to blocking the formation of AFs (28). Similarly, Green tea PPs in the concentration of 70 mg/ml suppressed the formation of AFs without side effects on the mycelial growth of APF (524). Phenolics also showed potential inhibitory profile against the production of other mycotoxins. In an interesting study, Boonmee and colleagues reported that simple hydroxycinnamic acid derivative, ferulic acid inhibited the production of OTA in *A. westerdijkiae* and *P. verrucosum* by 35 and 75% (525). Synthetic derivatives of flavonoids such as 5,6-dihydroxy-flavone and 5,6-dihydroxy-7-methoxy-flavone, in the concentration of 25 and 50 μg/ml, have significantly decreased the production of OTA in *A. carbonarius* after 8-day incubation (526). Similar to these outcomes, Romero et al. reported that caffeic acid, rutin and quercetin in the concentration of 250 mg/L remarkably decreased the production of OTA in *A. carbonarius* (527). Indeed, higher concentrations of these PPs (500 mg/L) completely inhibited the growth of OTA-producing fugus (527). The aqueous seed extract of *Trachyspermum ammi* also showed beneficial effects in degrading AFB1 mycotoxin. The phytochemical analyses revealed that *T. ammi* has different types of metabolites such as PPs, alkaloids, tannins, and other well-known natural substances (528).

Olive mill wastewater (OMWW) pure PPs such as caffeic acid, hydroxytyrosol, tyrosol and verbascoside also showed a decrease of nearly 99% in AFB1 production but had not influenced *A. flavus* growth (512). Accordingly, OMWW extract in the concentration of 15% was also decreased the formation of AFB1 ranged from 88 to 100%, respectively (512). In a dose-dependent manner, flavonoids-rich spent coffee grounds extract (PSCGE) has also found to be effective in degrading AFs (B1/2-G1/2) and OTA *in vitro* (529). The PSCGE was remarkably decreased the growth of toxicogenic fungi such as *A. flavus* and *A. ochraceus* as well as *Fusarium* species (529). These outcomes demonstrated that food wastes/residues have considerable level of health promoting metabolites; alternatively, can be used as potent inhibitors of APF and the production of AFB1 and other mycotoxins (529). Therefore, it should be noted that these waste by-products are trustworthy candidates to develop and formulate modified extracts with added values as anti-fungal agents.

Compared to PPs-rich extracts, alkaloids-rich extracts (ALEs) showed potential inhibitory properties in detoxifying of AFB1. In this respect, vasaka (*Adhatoda vasica* Nees) leaf ALEs displayed functionality to degrade AFB1 up to ≥98% after 24 h incubation at 37°C (530). The amide alkaloid piperlongumine isolated from *Piper longum* L. in the concentration of 0.2%w/v inhibited the biosynthesis of AFB1 in *A. flavus* up to 96% (531). Another piperidine alkaloid, piperoctadecalidine isolated from *P. longum* displayed 100% inhibitory profile against biosynthesis of AFB1 in the concentration of 0.7%, respectively (531). Other relevant studies also reported that ALEs are promising anti-fungal agents to prevent the formation of AFB1 and APF growth (532). It seems that the anti-aflatoxigenic property of alkaloids is dose-dependent, and the chemical variation of alkaloids might determine their inhibitory profile. In this regard, it can be said that both alkaloids/PPs-rich extracts and pure metabolites are influential compounds in the prevention of AFs; however, their chemistry and concentration are two determinant factors in defining their inhibitory/biological profile. In modern food industries, however, these metabolites are promising candidates to develop antifungal agents. These results together permit to harness phytochemicals for dealing with health hazardous mycotoxins.

## PPs-AFs related patents

Chemical fungicides (ChFs) are presently recruited for large-scale inhibition of AFs production and APF growth (533). However, as detailed in the literature, long-term application of ChFs may lead to ChFs-resistant APF (533) and the onset of health threatening symptoms (534). In this regard, there are only few registered patents publicly available to use PPs as candidate inhibitors of AFs biosynthesis. These innovations developed specific products or GM plants to prevent the spread of AFs-contaminated foods/crops. In an interesting patent, engineered transgenic plants with elevated levels polyphenol oxidase/gallic acid content showed resistance to *A. flavus* and AFs production (535). In another patent issued by Xiang et al., tea PPs in the concentrations of 0.2–1% were effective in the reduction of AFs biosynthesis by 21–81% (536). Dacheng et al. developed an anti-mycotoxigenic feed additive for cattle in which tea PPs (1–5 parts of whole patent formulation) have been used as main ingredients of this product (537). In another patent, a preparation method for constructing catechin nanoparticles has been suggested that could decrease the bioavailability of AFB1 and prevent hepatic injury (538). Despite the scarcity of patents on PPs for inhibition of AFs production, however, in the light of discussed materials herein further formulation of PPs can be crafted to develop potent anti-AFs products.

## Current status and future perspectives

Increasing knowledge on the antioxidant content of natural products and vitamins leads to developing “*antioxidant therapy*” to relieve human diseases (539), though there have been doubts in this field (540, 541). Studies have shown that the combination of PPs and vitamins enhances their antioxidant activity (539). It is now proven that cancer, MetSys and neurodegenerative diseases are associated with a higher level of oxidative/nitrosative stress and ROS/RNS production (30, 542–544). In this regard, natural antioxidants are primary defensive agents in preventing early phases of cancer, AD and DM progression (30, 388, 545). Accordingly, PPs received a huge volume of attention due to their antioxidant properties and becoming the relevant metabolites in antioxidant therapy programs (546). Presently a considerable number of PPs in the form of antioxidant supplements, cosmetic and food additive products, sanitary agents, antibacterial products, and pain relievers are available in global markets for non-clinical applications (338).

Regarding PPs applications in alleviating AFs-induced health challenges, there are several key points should be highlighted before consideration of these metabolites for large-scale studies. First, despite considerable studies conducted on PPs, the optimum doses of phenolic compounds for amelioration of AFs side effects have not yet determined. This case causes a significant variability in observed biological effects assigned to PPs. For example, studies have shown that the anticancer activity of PPs is dose-dependent, in which PPs shared different IC50 and Ki values for inhibition of target enzymes (547). On the other hand, the intake of high concentration of PPs might show adverse effects on kidney and thyroid hormones level, as reported in animal models (548, 549) and argued in the literature (540).

Second, standardization of PPs mode of action toward cellular receptors requires sufficient data generated from clinical trials and large-scale studies (546). For instance, the anticancer activity of PPs depends on their chemical structure, doses and subtypes of cancers (399). Third, as detailed in Table 1, anti-AFs properties of PPs are not generally focused on specific pathways, though the majority of studies confirmed that these metabolites function through suppressing of AFs-induced oxidative stress. This demonstrated that PPs might show off-target effects to interact with several different receptors in the body. Fourth, the pro-oxidant activity of PPs is another concern (547, 550) might delay the recruitment of these metabolites against AFs. On the other hand, some phenolic compounds such as daidzein and genistein exhibited controversial effects on the pathogenesis of hormone-associated cancers (399). Such incongruous results have still not been reported for other phenolic categories, and it is now believed that PPs displayed their health-promising effects by interacting with various receptors and signaling pathways (551). In this respect, the emerging scientific investigations reported that supplementation of antioxidants (vitamin E and N-acetylcysteine) in mice increased the progression of lung cancer by inducing P53-assisted oxidative stress (198). This finding suggests that excessive supplementation of antioxidants might increase the progression of nCDs, therefore, to avoid further complications, and for cautionary reasons, the consumption of antioxidants should be followed by considering optimum doses under strictly controlled condition. Therefore, recruiting PPs or PPs-rich extracts in anti-AFs therapies requires a deep insight into their bioavailability, on-target mode of action, pharmacokinetics, drug interventions, and chemical stability in the human body (188, 547).

Another worthy point that should be addressed that is PPs-rich waste products (e.g., OMWW) disclosed potential inhibitory activity against AFB1 formation (512). Such products have a certain group of PPs, called non-extractable PPs (NEPPs) (552, 553), in turn, their biological activities have not extensively been investigated due to limitations in extraction methods or insolubility and polymeric essence of these metabolites (554). Studies have shown that the optimization of PPs extraction methods using innovative technologies reduced required time and energy to elucidate PPs, and improved the quantity of achievable phenolics metabolites (555). These innovations help researcher to access the whole PPs profile of herbaceous materials to determine their biological activities. In this regard, we previously reviewed the valorization methods of OMWW PPs extraction to highlight the biological benefits of these phenolics that widely released into the nearby environment (556). Similar studies also suggested that such NEPPs from waste products have a remarkable antioxidant content, resultantly may exhibit health promoting effects in the cornerstone of the human diseases prevention (557).

On the other hand, the current knowledge of PPs is mainly associated with extractable *O-*glycosidic derivatives. The evidence purported that C-glycosidic PPs such as schaftoside derivatives exhibited potential biological activity to enhance crop resistance against pests (558). Therefore, such metabolites can also be used to inhibit the growth of APF or prevent the production of AFs.

**Figure 12:**
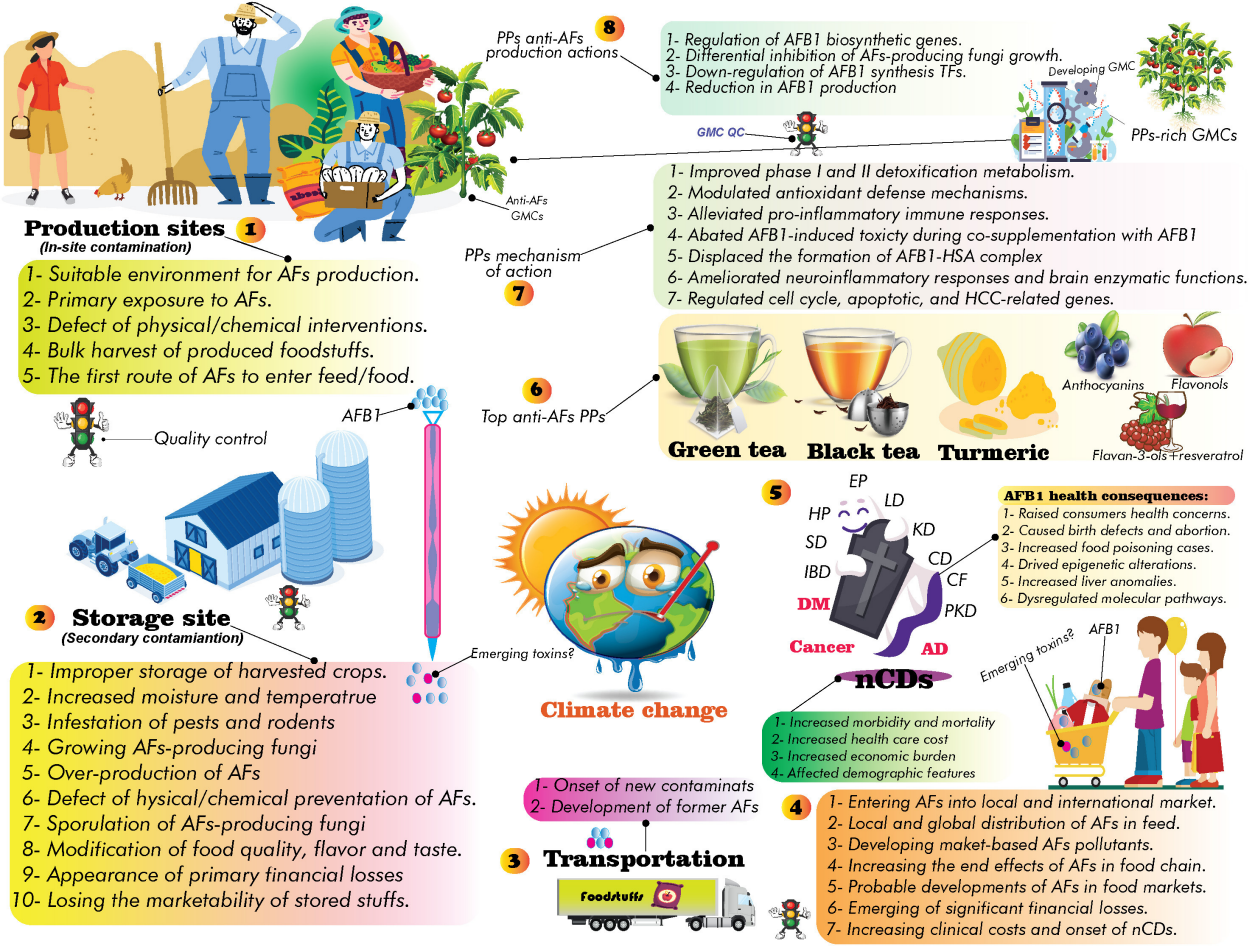
The findings of this study at a quick glance. AFs in production/storage sites caused damage to target products and passed to consumers, leading to the progression of nCDs (Steps 1–5). Certain classes of PPs alleviated the side effects of AFs by modulating several critical signaling pathways involved in the pathogenesis of AFs. Indeed, these compounds could inhibit the formation of AFs by regulating expression of critical genes involved in biosynthetic pathways of AFs (Steps 6–8). Climate change markedly affects the occurrence of AFs, therefore, developing PPs-rich GM plants might be considered as a strategic policy to decrease the quantity of these mycotoxins. GMCs, genetically modified crops; PKD, Parkinson's disease; EP, epilepsy; IBD, inflammatory bowel disease; CD, cardiovascular diseases; KD, kidney diseases; LD, liver diseases; HP, hemophilia; SD, seizure disorder; CF, cystic fibrosis.

Additionally, the current findings on anti-AFs activity of PPs obtained from animal models (rodents and chicken) and experimental assays, in turn, requiring further validations. Future studies on how PPs might interact with nCDs-associated molecular receptors and signaling pathways (in particular inflammation) and their exact mode of action in relieving AFs end effects should be conducted to confirm their efficacy and safety for medical management of mycotoxins. Figure 12 summarized the current findings of this study.

## Concluding remarks

Our literature review clearly manifested that shifts in world climate can influence the distribution/quantity of AFs and prevalence of nCDs. Additionally, the triangle of GCC/exposure to AFs/progression of nCDs has significant complications for human health, in turn, can increase the economic costs to countries health care system. It is important to conduct more large-scale and long-term investigations in susceptible countries to GCC (in particular low and middle-income regions) to predict future threats for designing effective preventative policies and health risk assessments. Various types of food/feed items have been identified with exceeded level of AFs contaminants, resultantly this phenomenon might bring countries more deaths during the upcoming years owing to the onset of nCDs complications. The co-occurrence of other GCC-associated risk factors may expedite the progression of nCDs, though this claim requires further approvement to know how synergistic effects of environmental health hazardous risks will threaten human health and lifestyle.

Countries implemented rigorous regulatory measurements to monitor and diagnosis of AFs in contaminated foods/feeds. However, the literature indicates that the current regulatory settings should immediately be revisited due to the advent of AFs and other emerging mycotoxins in various food commodities (559, 560). AFs decreased the quality and marketability of food/feed products, therefore causing damages to countries food safety and economy (561). In this regard, increasing public awareness, in particular among farmers and local food/feed producers, plays a critical role in elimination of AFs and preparing countries local society to deal with complications of GCC and health threatening environmental risk factors.

Owing to the role of AFs in triggering immunosuppression (37), interfering with protein metabolism and micronutrient deficiency (276), and reducing antibody production (562), specific diagnostic/predictive biomarkers should be characterized in early detection of AFs-induced health challenges to a better management of clinical symptoms. On the contrary, PPs promoted initiate immune responses by activating certain signaling pathways (563). This indicates that dietetic intervention using PPs is an effective way to modulate immune responses to alleviate the toxicity of AFs, though current PPs gaps for clinical applications should be addressed in detail. In neurodegenerative diseases, the accumulation of aggregated proteins leads to the progression of these disorders (30, 564). PPs have also shown regulatory effects in the modulation of protein metabolism and activation of protein degrading systems to prevent the accumulation of misfolded proteins (564). Having such biological properties enabled PPs to combat AFs health problems.

As discussed, no antidote has been introduced to alleviate the toxicity of AFs and current management scenarios of AFs complications are based on the removal of these mycotoxins in foods. Indeed, the frequency of international studies on AFs has spectacularly surged up in the past years in which the USA, China, India occupied the top ranks of studies in this field. Bibliometric analysis of the literature displayed that the majority of studies conducted on AFs were focused on carcinogenic, properties and detection methods of these toxins, though association of exposure to AFs and the onset of DM and AD has been remarkably taken into consideration. This indicates that long-term exposure to AFs evoked multidimensional health challenges in addition to their potential in inducing HCC.

The literature reviewed herein suggested that the quest for characterization of natural inhibitors of AFs is becoming a global trend in food safety field. According to our literature review, phenolics and PPs-rich extracts are promising AFs detoxifying products. Green and black tea, turmeric, anthocyanins and flavonols were the most studied phenolics metabolites to detoxify AFs complications in animal and *in vitro* studies. Despite the lack of enough clinical data on the effectiveness of PPs in preventing AFs consequences, the available data displayed that PPs showed a heterogenous biological activities in preventing the side effects of AFs by targeting several different molecular receptors. PPs reviewed in this paper can be used for decontamination of AFs (and possibly other emerging mycotoxins) either in the human/animal body or production/storage sites after considering safety cautions.

As discussed, due to the negative effects of nCDs and GCC on world economy, future studies should seek to develop strategies that may improve the bioavailability, mode of action, and pharmacokinetics properties of PPs in the cornerstone of AFs-induced nCDs treatment. Coupling antioxidant-assisted interventions with conventional physio-chemical removal procedures of AFs in food/feed items and developing GM PPs-rich crops are highly recommended to decrease the quantity of AFs to lowest concentrations and improve lifestyle and longevity of affected individuals. It is also treasured to address this point that regular consumption of PPs or PPs-rich functional foods might help in early preventing of AFs-induced nCDs, though this finding requires further clinical assessments.

## Author contributions

HR designed the study, wrote the first draft, and prepared the graphical illustrations. HR and FN conducted a literature search. RK revised the draft. HR, FN, and RK revised the final draft. All authors contributed to the article and approved the submitted version.

## Conflict of interest

The authors declare that the research was conducted in the absence of any commercial or financial relationships that could be construed as a potential conflict of interest.

## Publisher's note

All claims expressed in this article are solely those of the authors and do not necessarily represent those of their affiliated organizations, or those of the publisher, the editors and the reviewers. Any product that may be evaluated in this article, or claim that may be made by its manufacturer, is not guaranteed or endorsed by the publisher.

## Abbreviations

PPs: phytophenolics or polyphenols
HSA: human serum albumin
AD: Alzheimer's disease
DM: diabetes mellitus
nCDs: non-communicable diseases
AFs: aflatoxins
GMO: Genetically modified organisms
GM: genetically modified
N-flavonoids: non-flavonoids
SOD: superoxide dismutase
OTA, Ochratoxin A, T1D: type 1 DM
T2D: type 2 DM
EU: European union
US: United states
IARC: international agency for research on cancer
ROS: reactive oxygen species
RNS: reactive nitrogen species
BBB: the blood brain barrier
CNS: central nervous system
NIF: neuroinflammation
AFB1-NIF: AFB1-induced neuroinflammation
IL: interleukins
TNF: tumor necrosis factor
IGF2: insulin-like growth factor-2
IGF-IR: IGF1 receptor
HCC: hepatocellular carcinoma
MetSys: metabolic syndromes
WHO: world health organization
GSH: glutathione
GST: glutathione-S-transferase
GSH-Px: Glutathione peroxidase
MDA: Malondialdehyde
AST: aspartate aminotransferase
ALT: alanine aminotransferase
GGT: gamma glutamyl-transferase
KCs: Kupffer cells
INF-γ: Interferon gamma
TLRs: toll-like receptors
MAPK: mitogen-activated protein kinase
MyD88: myeloid differentiation primary response 88
CxCR4: C-X-C chemokine receptor type 4
PI3K: phosphoinositide 3-kinases
AKT: protein kinase B
COX-2: prostaglandin-endoperoxide synthase 2
iNOS: inducible nitric oxide synthase
mTOR: mammalian target of rapamycin
Nrf2: nuclear factor erythroid 2–related factor 2
HO-1: Heme oxygenase-1
STAT: signal transducer and activator of transcription
AMPK: 5&#8217
AMP-activated protein kinase
JAK: Janus tyrosine kinase
NF-κB: Nuclear factor kappa B
CCK: cerebral creatine kinase
PKC: protein kinase C
CAT: catalase
LDH: lactate dehydrogenase
TNFRs: TNF-α receptors
CK: creatine kinase
AP: acid phosphatase
AChE: acetylcholinesterase
ADA: adenosine deaminase
*p-*NF-κB: phosphorylated NF-κB
CYP: Cytochromes P450
HBC/V: hepatitis B/C virus
PPI: protein-protein interaction
HPLC: High-performance liquid chromatography
IAC: immunoaffinity chromatography
LC-MS/MS: Liquid Chromatography with tandem mass spectrometry
HPLC/FLD: HPLC with fluorescence detector
LFIA: Lateral flow immunoassay
ME: metabolic engineering
PBPs: PPs biosynthetic pathways
PAL: phenylalanine ammonia lyase
MMPs: Matrix metalloproteinases
EGFR: epidermal growth factor receptor
ERK: Extracellular signal-regulated kinase
FOX: fork-head box
ALK: Anaplastic lymphoma kinase
JNK: c-Jun N-terminal kinase
VEGF: Vascular endothelial growth factor
ROS1: Proto-oncogene tyrosine-protein kinase
PPAR: peroxisome proliferator-activated receptors
IDO, Indoleamine 2: 3 dioxygenase
TIMP3: TIMP Metallopeptidase Inhibitor 3
PDK: Phosphoinositide-dependent Protein Kinase
MUP1: major urinary protein 1
GCC: Global climate change
GW: global warming
APF: AFs-producing fungi.
