# Supplementary material for: May phytophenolics alleviate aflatoxins-induced health challenges? A holistic insight on current landscape and future prospects
Source: Front Nutr. 2022 Oct 28;9:981984. doi: 10.3389/fnut.2022.981984 (PMC9649842; doi:10.3389/fnut.2022.981984)
Supplement: Supplementary file 1 [file Data_Sheet_1.docx]

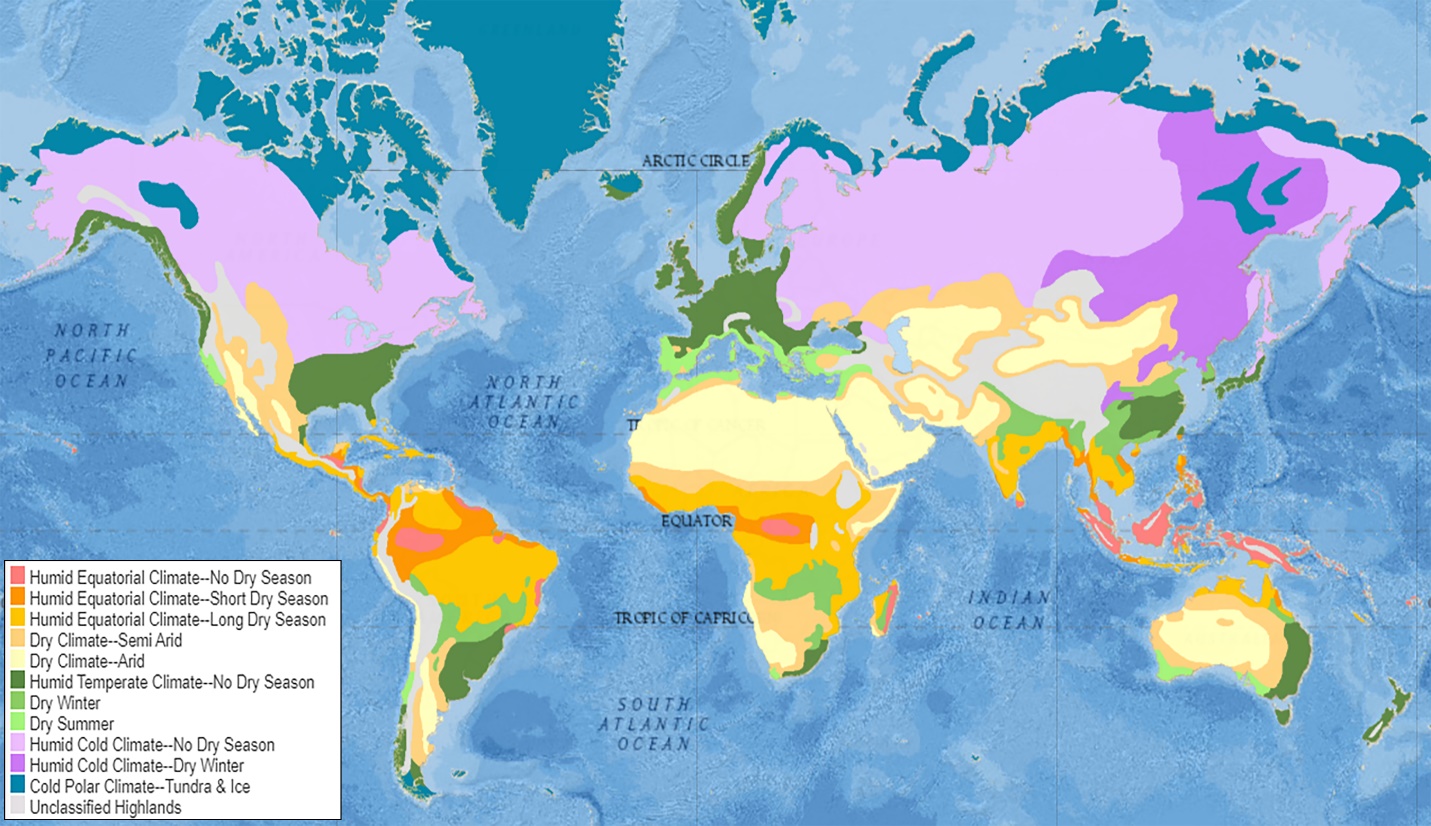


**Figure S1: distribution of world climate zones. Photo adopted from National geography website [1]**


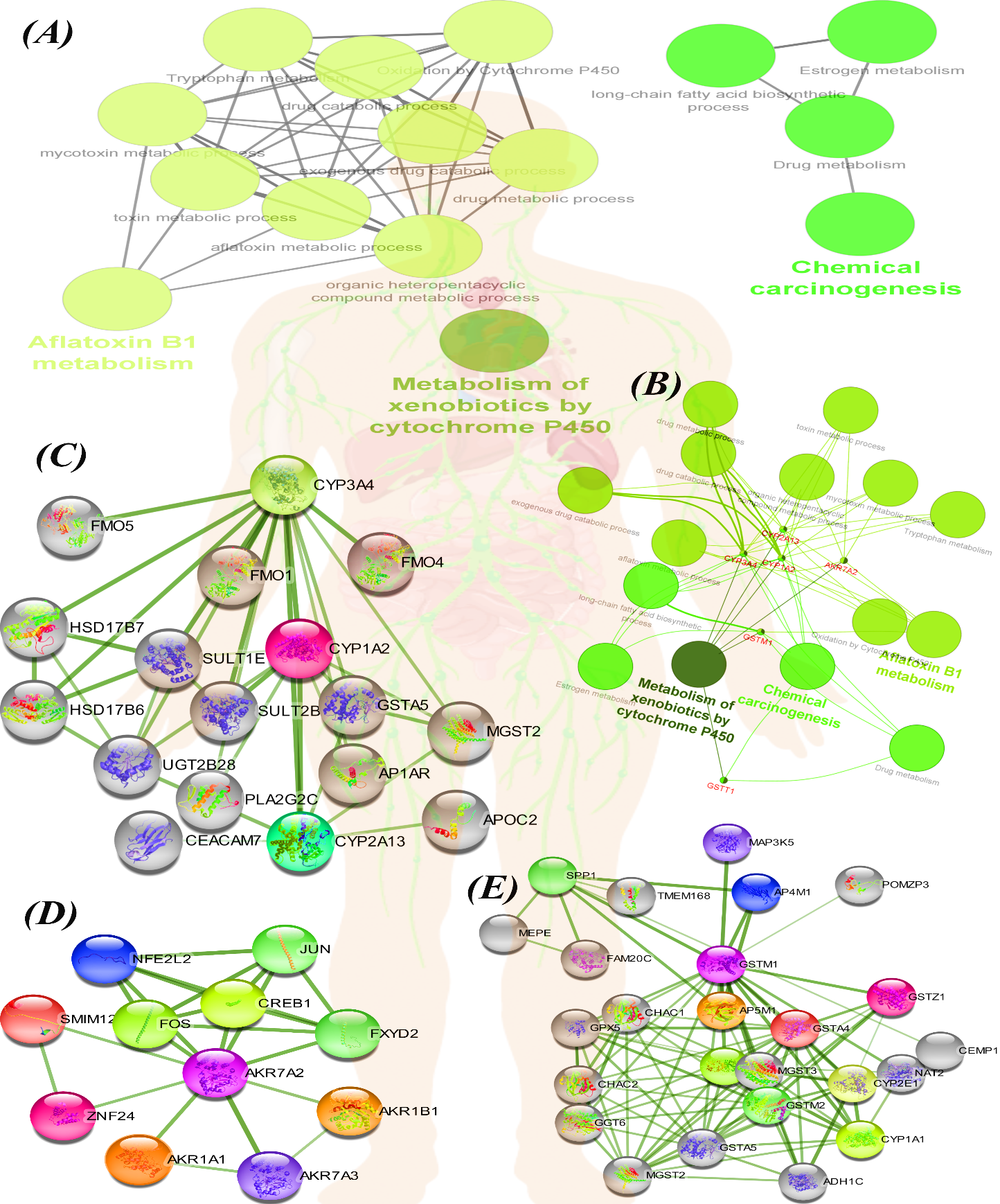


**Figure S1: Protein-protein interaction networks of genes involved in AFB1 metabolism. (A) the association of the AFB1 network and other xenobiotics metabolism pathways; (B) the interaction of genes involved in AFB1 metabolism with each other and relevant pathways; (C) the PPI network of CYP3A4 and CYP1A2 proteins involved in AFB1 metabolism; (D) PPI network of AKR7A2 protein; (E) PPI network of GSTM1. The thickness of lines determined the potential interactions of target proteins with each other or nearby proteins.**


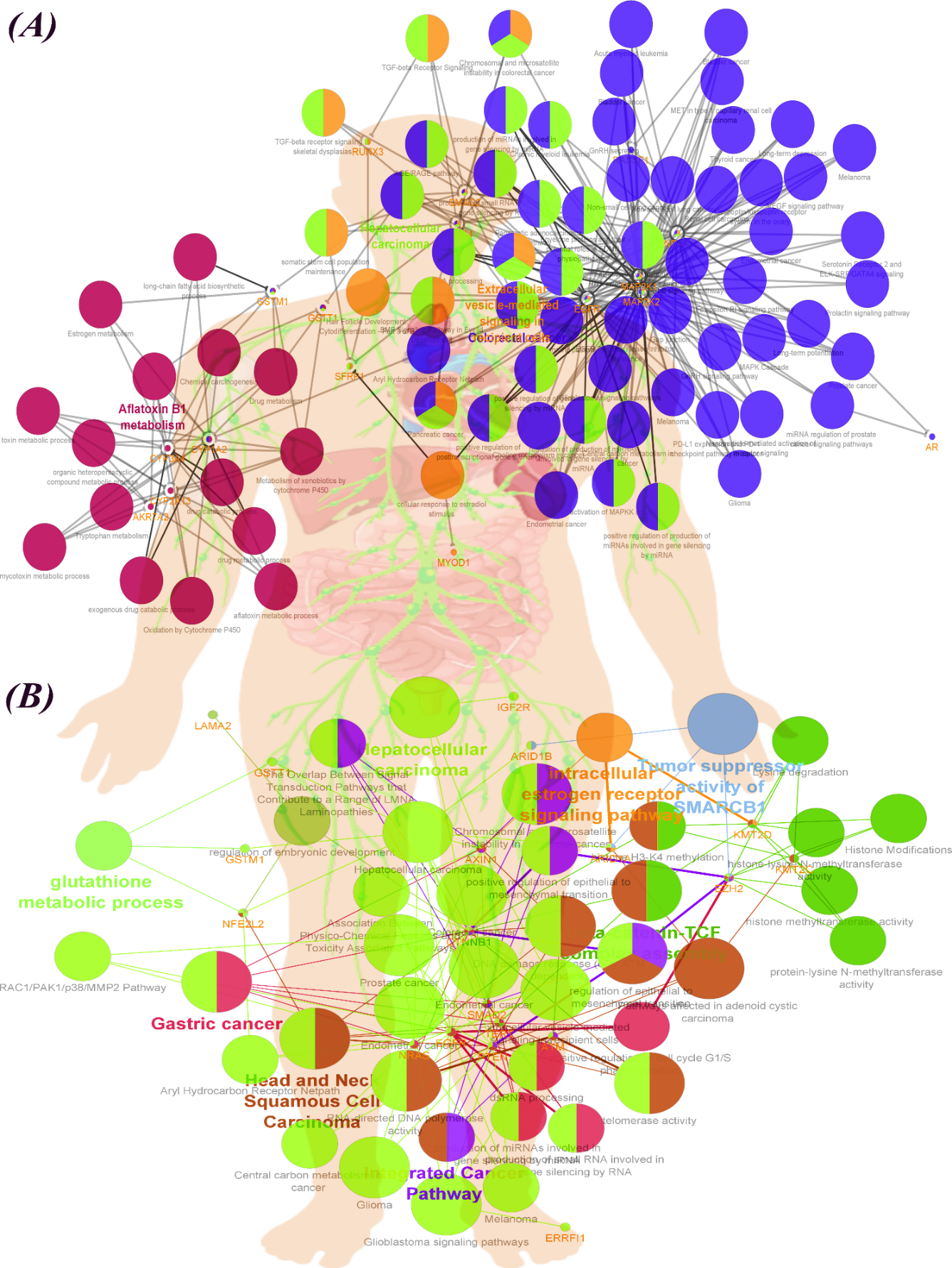


**Figure S3: The possible participation of AFB1 metabolism network in the pathogenesis of cancer and dysregulation of molecular components. (A) the association of AFB1 metabolism in the PPI network of liver, bladder, colorectal, thyroid, melanoma, and pancreatic cancer. (B) the detailed interactome network of AFB1 metabolism genes and critical signaling pathways and molecular components involved in the pathogenesis of cancers. The thickness of lines shows the higher possibilities for PPI. Circles with different colors display the multifunctional activity of target pathways.**

1. Geographic, N. *World climate zones*. 2022 [cited 2022 June 19, 2022]; Available from: <https://mapmakerclassic.nationalgeographic.org/hGBQ57UFwqfZ8ypD5cjGDx/#/>.
